# Supplementary material for: The Discovery of Indole-2-carboxylic Acid Derivatives as Novel HIV-1 Integrase Strand Transfer Inhibitors
Source: Molecules. 2023 Dec 8;28(24):8020. doi: 10.3390/molecules28248020 (PMC10745497; doi:10.3390/molecules28248020)
Supplement: Supplementary file 1 [file molecules-28-08020-s001.zip › molecules-2732503-supplementary.pdf]

The Discovery of Indole-2-Carboxylic Acid Derivatives as Novel HIV-1 Integrase Strand Transfer Inhibitors

Yu-Chan Wang, Wen-Li Zhang, Rong-Hong Zhang, Chun-Hua Liu, Yong-Long Zhao, Guo-Yi Yan, Shang-Gao Liao, Yong-Jun Li, Meng Zhou

Table of Contents

1. Supporting Figures and Tables

Table S1……………………………………………………………………………2

Table S2……………………………………………………………………………4

Figure S1……………………………………………………………………………5

2. ^{1}H and ^{13}C NMR spectra of target compounds ……………………………………… 7

3. IC_{50} and CC_{50} curves of target compounds ……………………………………… 21

Table S1. Libdock scores of 168 compounds through ADMET screening.

Number	HIT ID	Libdock score	Number	HIT number	Libdock score
1	HIT101170378	202.907	85	HIT100564457	173.910
2	HIT104892884	197.552	86	HIT105559345	173.795
3	HIT105209452	193.069	87	HIT211693403	173.791
4	HIT101131099	192.152	88	HIT105485118	173.788
5	HIT103374264	191.023	89	HIT105860908	173.777
6	HIT211718027	190.364	90	HIT103795749	173.383
7	HIT100492556	190.301	91	HIT102119074	173.186
8	HIT100469744	189.759	92	HIT100097824	173.186
9	HIT103895409	189.674	93	HIT101273521	172.760
10	HIT105002790	188.779	94	HIT101715391	172.729
11	HIT102760498	188.766	95	HIT100479963	172.717
12	HIT102249045	188.523	96	HIT106479088	172.646
13	HIT101578102	188.285	97	HIT102057128	172.446
14	HIT100351328	187.846	98	HIT211741150	172.368
15	HIT100924302	187.667	99	HIT105661843	172.337
16	HIT101706818	187.626	100	HIT102435924	172.230
17	HIT104972602	185.637	101	HIT105680324	172.119
18	HIT107217090	185.492	102	HIT211714541	172.109
19	HIT100364607	185.342	103	HIT100640440	171.817
20	HIT211739001	185.169	104	HIT101057010	171.700
21	HIT101487293	184.551	105	HIT105403058	171.494
22	HIT107340271	184.088	106	HIT107231803	171.452

23	HIT103034199	183.929	107	HIT105777966	171.352
24	HIT104636999	183.590	108	HIT100456990	171.210
25	HIT106288749	183.219	109	HIT106066563	171.164
26	HIT106179857	183.044	110	HIT101819288	170.968
27	HIT107477728	182.979	111	HIT104095482	170.907
28	HIT103738299	182.864	112	HIT105765957	170.878
29	HIT105858750	182.525	113	HIT105454002	170.865
30	HIT104550697	182.255	114	HIT105650628	170.572
31	HIT107730373	182.056	115	HIT101815181	170.287
32	HIT107076057	181.658	116	HIT105253650	170.197
33	HIT103111053	181.361	117	HIT100844343	169.990
34	HIT104797126	181.352	118	HIT103951514	169.977
35	HIT105686390	181.316	119	HIT100160861	169.702
36	HIT101038334	181.252	120	HIT103457531	169.627
37	HIT105381385	180.848	121	HIT100396312	169.450
38	HIT102385644	180.269	122	HIT103142521	169.425
39	HIT107130254	180.242	123	HIT100720519	169.276
40	HIT100294707	180.183	124	HIT106725747	169.175
41	HIT101057921	180.141	125	HIT105697825	169.172
42	HIT100850905	179.762	126	HIT105690751	169.130
43	HIT106570151	179.657	127	HIT102179127	169.056
44	HIT211521964	179.569	128	HIT211703833	169.017
45	HIT105386960	179.534	129	HIT100604561	168.896
46	HIT103039373	179.369	130	HIT106806985	168.851
47	HIT10369678	179.201	131	HIT211723371	168.818

	9				
48	HIT211718024	178.958	132	HIT107706176	168.813
49	HIT211449711	178.438	133	HIT103811857	168.609
50	HIT102164670	177.547	134	HIT211470174	168.462
51	HIT100456495	177.473	135	HIT101225492	168.419
52	HIT211683095	177.261	136	HIT105062290	168.363
53	HIT102639309	177.094	137	HIT212976827	168.356
54	HIT103123013	176.971	138	HIT105250832	168.299
55	HIT102248529	176.636	139	HIT100565319	168.296
56	HIT105403256	176.630	140	HIT106924020	168.270
57	HIT100310720	176.579	141	HIT101301506	168.240
58	HIT107227353	176.578	142	HIT107729566	168.038
59	HIT101217035	176.219	143	HIT100252391	168.023
60	HIT103500970	176.115	144	HIT102924146	167.934
61	HIT211733493	176.099	145	HIT103588978	167.922
62	HIT105773305	176.054	146	HIT211483803	167.863
63	HIT106457745	176.004	147	HIT107637279	167.849
64	HIT211364982	175.858	148	HIT105346675	167.783
65	HIT101947095	175.671	149	HIT105080951	167.678
66	HIT101973378	175.635	150	HIT106620668	167.568
67	HIT106037793	175.435	151	HIT104623204	167.534
68	HIT100730776	175.385	152	HIT211719041	167.376
69	HIT100764658	175.332	153	HIT100811644	165.329
70	HIT103901995	175.247	154	HIT100081044	163.024
71	HIT105551656	175.033	155	HIT105050727	162.267

72	HIT100744403	175.024	156	HIT103408850	162.112
73	HIT101894868	174.900	157	HIT107232144	160.597
74	HIT101490558	174.854	158	HIT100661494	160.191
75	HIT104549290	174.796	159	HIT100651577	160.165
76	HIT105835288	174.468	160	HIT107093086	159.583
77	HIT105477401	174.377	161	HIT102016638	159.455
78	HIT212980596	174.180	162	HIT103374703	158.472
79	HIT103684232	174.130	163	HIT105499167	158.307
80	HIT100007538	174.128	164	HIT103778891	158.125
81	HIT106927617	174.100	165	HIT102037016	158.086
82	HIT100561799	174.086	166	HIT100277761	157.662
83	HIT103280811	174.061	167	HIT105702772	157.608
84	HIT106981961	173.935	168	HIT101037707	157.413

Table S2. Binding free energies (kcal·mol^{-1}) of 25 compounds through autodock vina.

Number	HIT ID	Lowest binding energy (kcal/mol)	Highest binding energy (kcal/mol)	Most binding energy
1	HIT105080951	–18.0	–12.2	–18.0
2	HIT103034199	–16.2	–12.0	–16.2
3	HIT101057921	–15.8	–11.4	–15.8
4	HIT101057010	–15.7	–7.8	–10.3
5	HIT104550697	–15.3	–11.3	–15.25
6	HIT104315479	–15.1	–14.5	–15.1
7	HIT100844343	–14.4	–11.4	–14.4
8	HIT101894868	–14.2	–11.4	–14.2
9	HIT105050727	–14.2	–11.9	–13.2
10	HIT211521964	–14.1	–8.2	–12.8
11	HIT100561799	–13.9	–10.5	–13.9
12	HIT103374264	–13.7	–10.2	–13.7
13	HIT100364607	–13.7	–8.1	–12.4
14	HIT103895409	–13.6	–10.6	–13.6
15	HIT107076057	–13.5	–8.8	–13.5
16	HIT100811644	–13.4	–11.0	–13.4
17	HIT104892884	–13.4	–8.6	–13.4
18	HIT211449711	–13.3	–8.2	–13.3
19	HIT106924020	–13.2	–8.8	–13.2
20	HIT106570151	–13.2	–12.3	–13.2
21	HIT105485118	–13.2	–11.7	–13.1
22	HIT105499167	–13.2	–9.4	–11.9
23	HIT106066563	–13.1	–8.5	–12.7
24	HIT101131099	–13.1	–9.7	–11.7
25	HIT102924146	–12.9	–11.4	–12.9

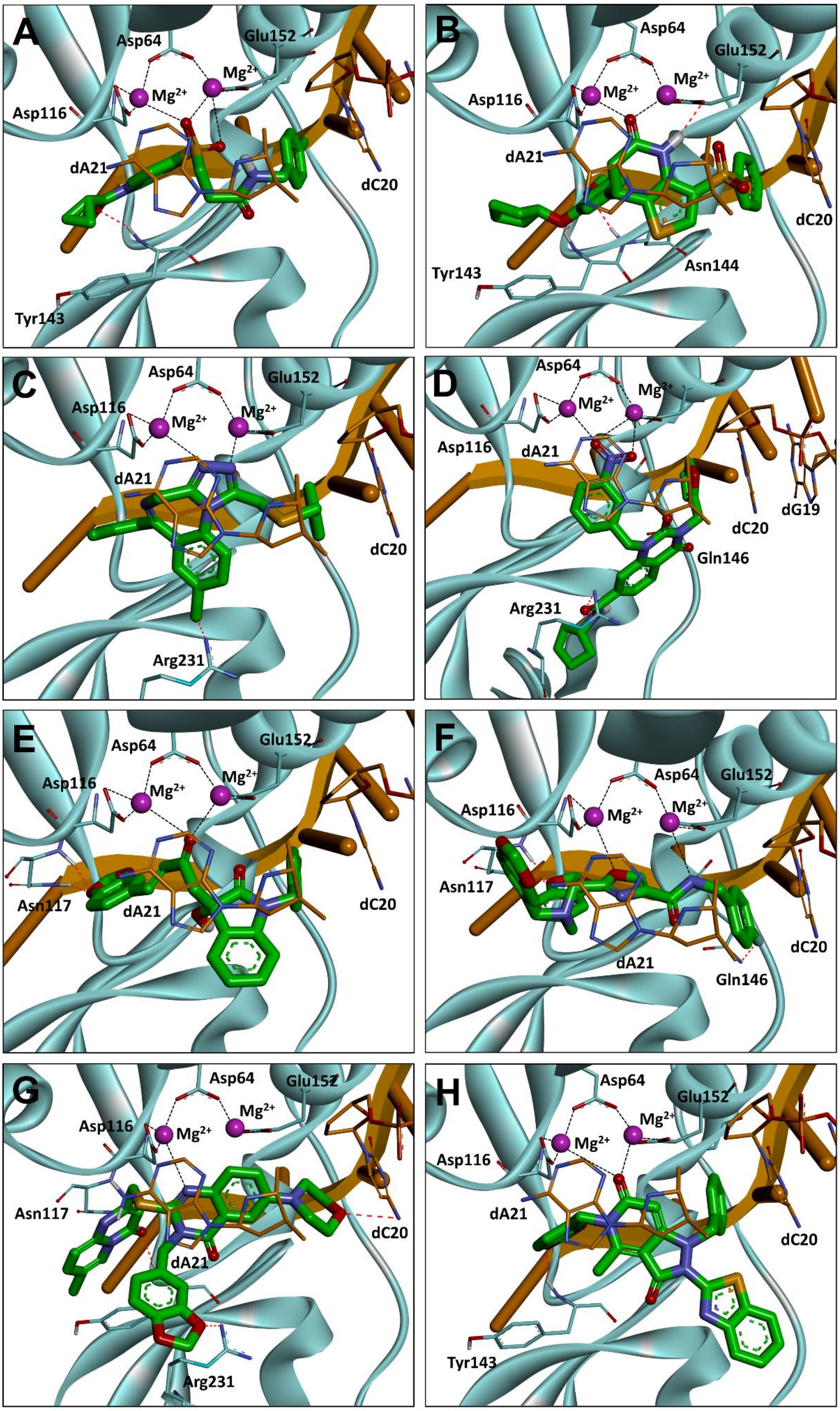

Figure S1. Binding mode analysis of HIT101057921 (A), HIT104550697 (B),

HIT106924020 (C), HIT105485118 (D), HIT105499167 (E), HIT106066563 (F), HIT101131099 (G), and HIT102924146 (H) with HIV-1 integrase (PDB ID: 6PUY). HIV-1 integrase is displayed in cyan, the 3′ end of the viral DNA (dA21 and dC20) is shown in stick representation in orange, and the chelate bond and hydrogen are represented with dashed line in black and red, respectively.

Spectral copies of ^{1}H NMR and ^{13}C NMR of compounds

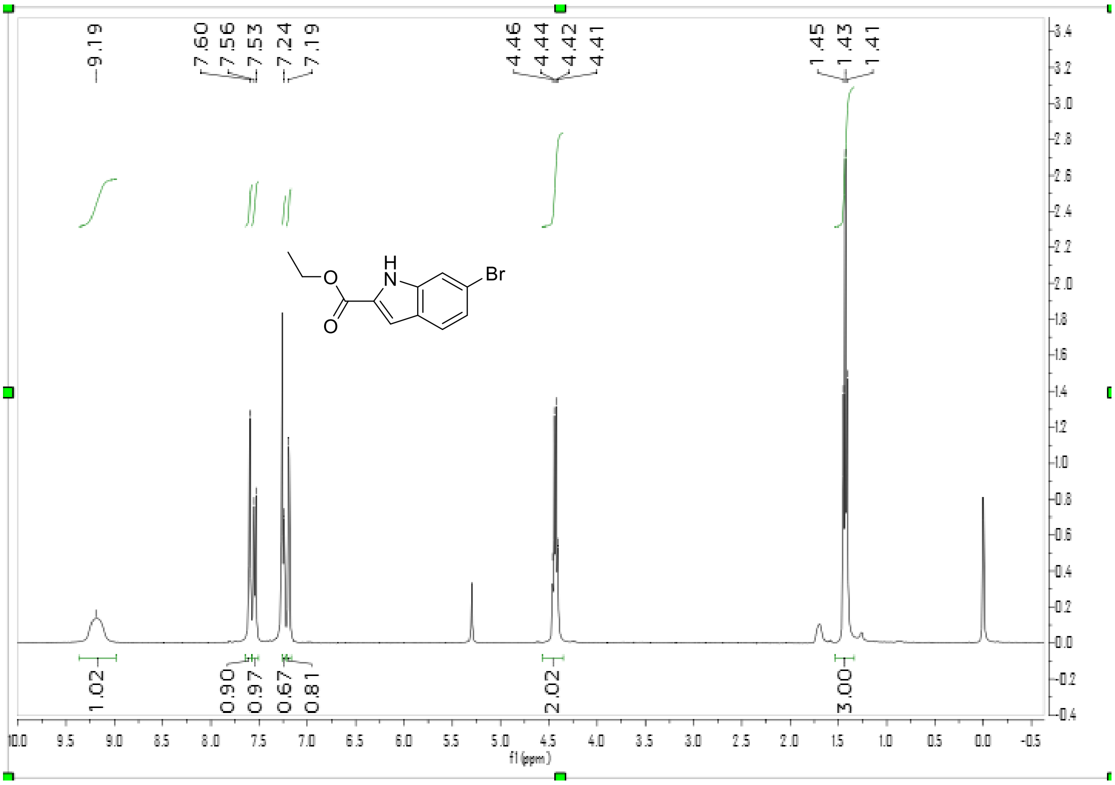

^{1}H NMR spectrum of **12**

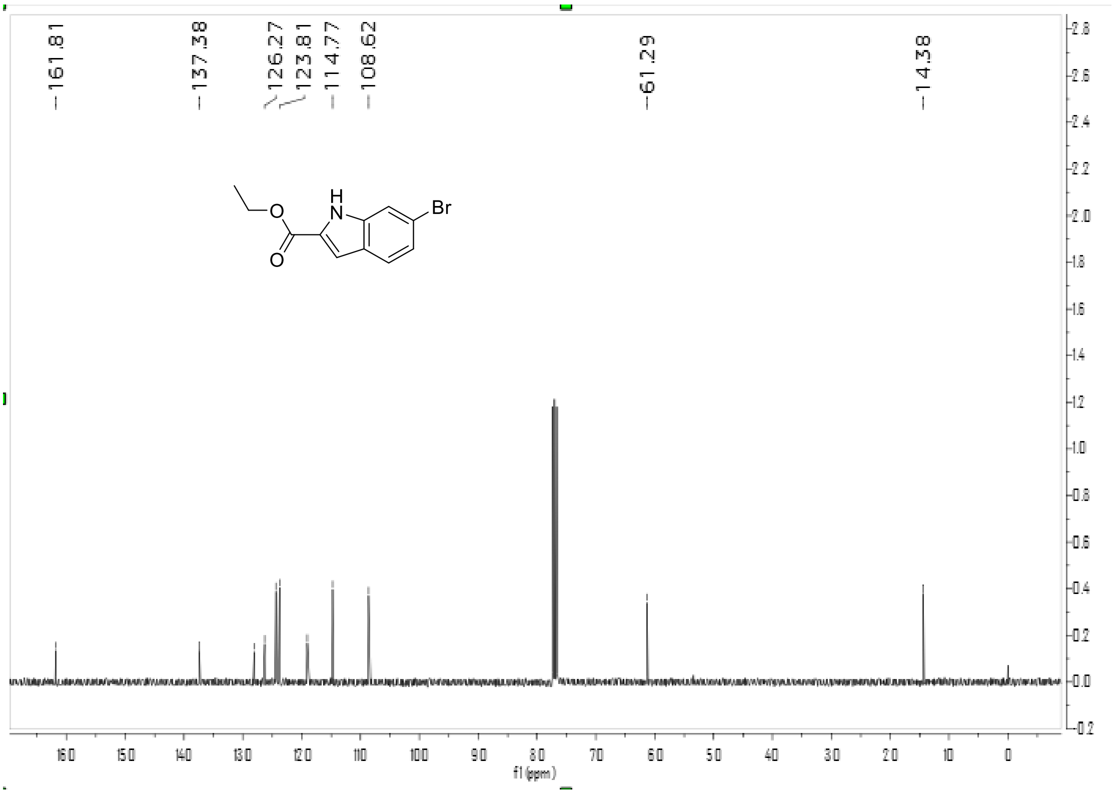

^{13}C NMR spectrum of **12**

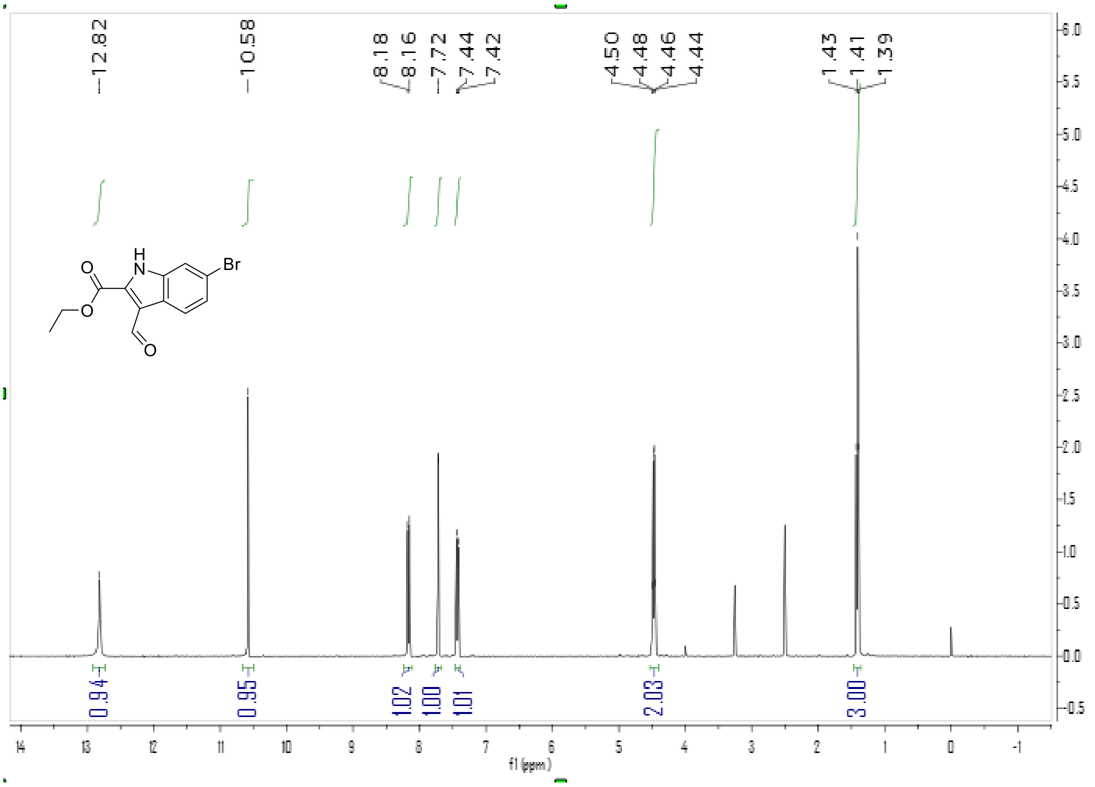

^{1}H NMR spectrum of **13**

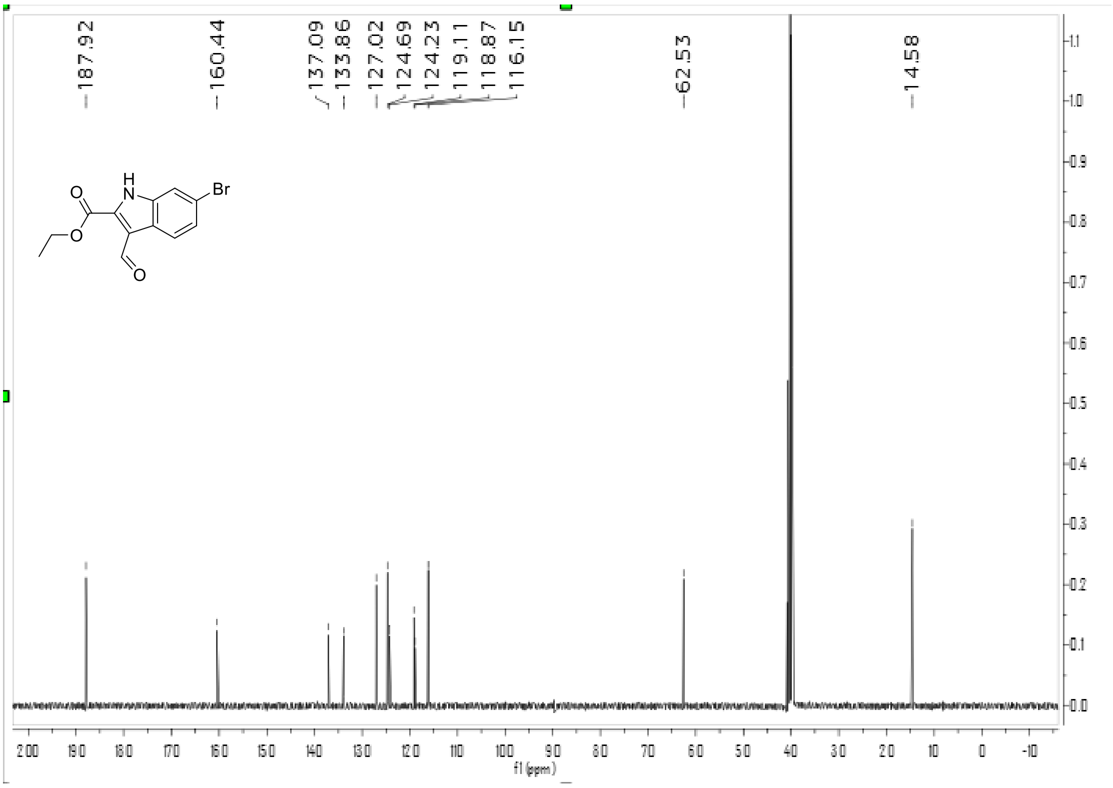

^{13}C NMR spectrum of **13**

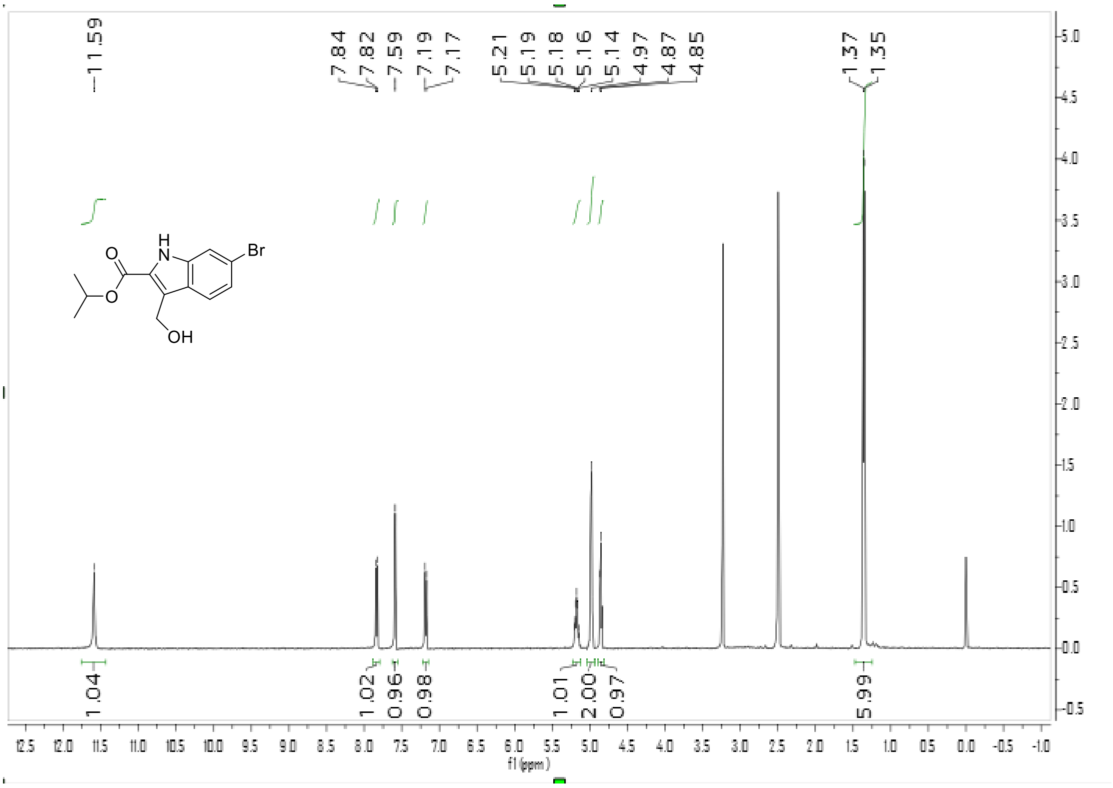

^{1}H NMR spectrum of **14**

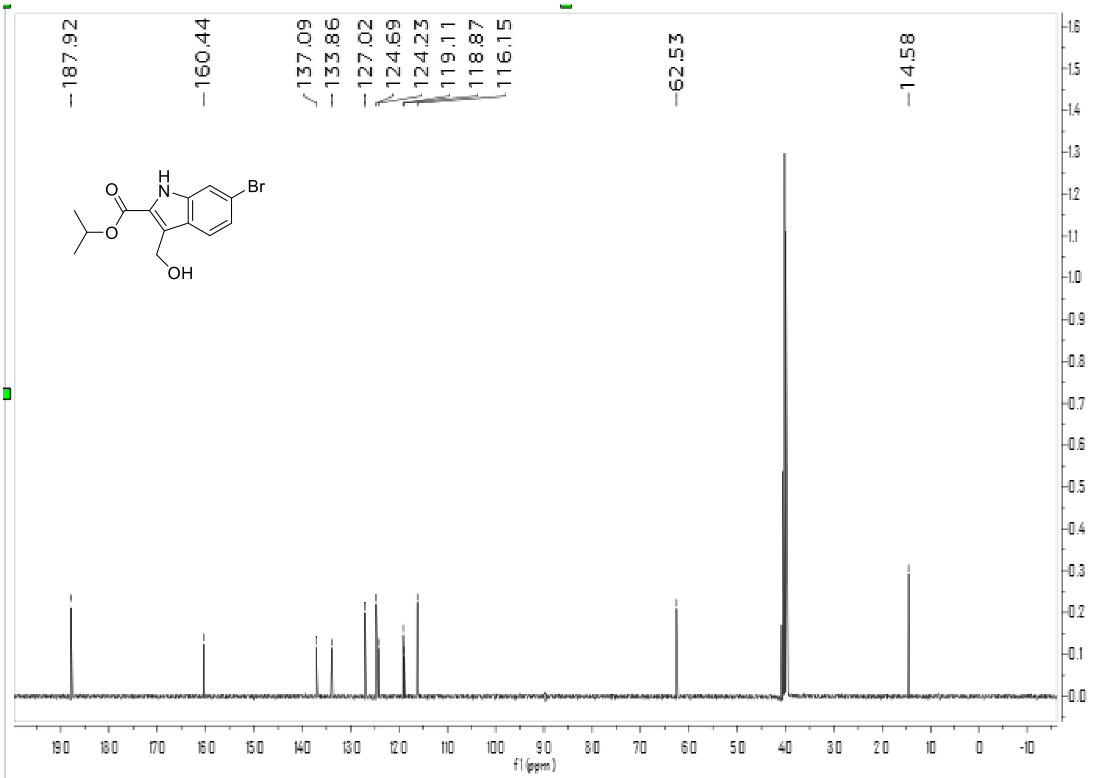

^{13}C NMR spectrum of **14**

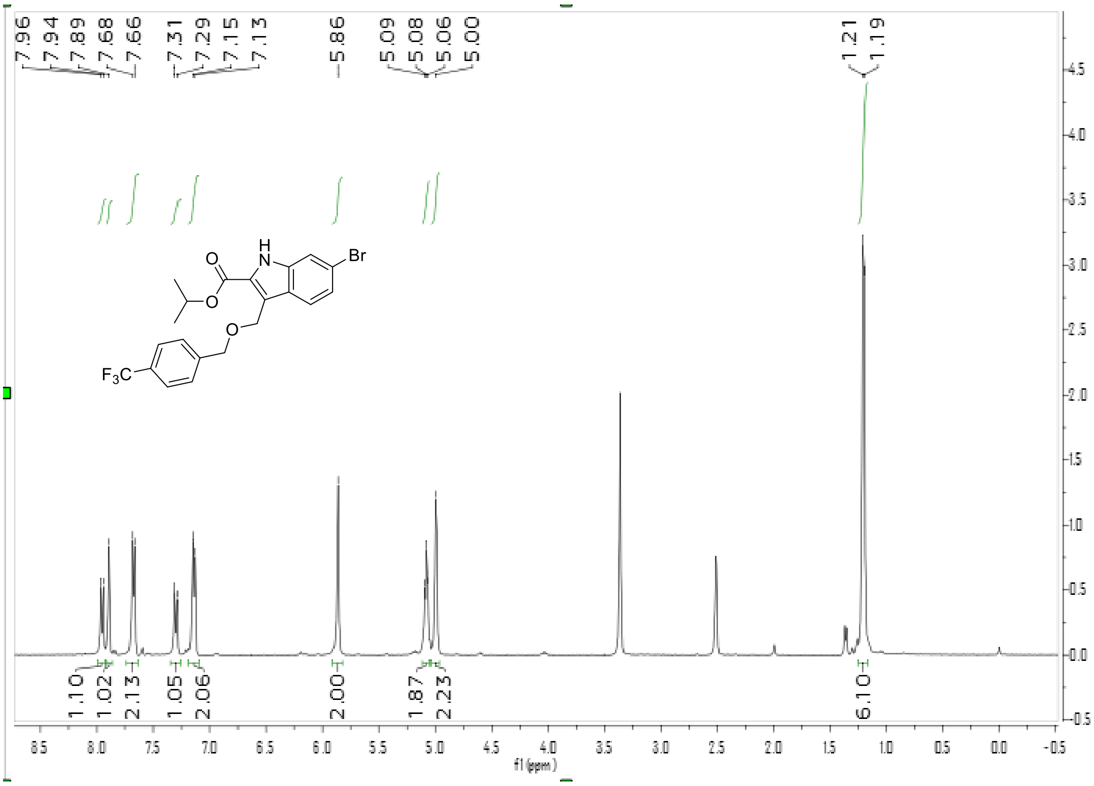

^{1}H NMR spectrum of **15**

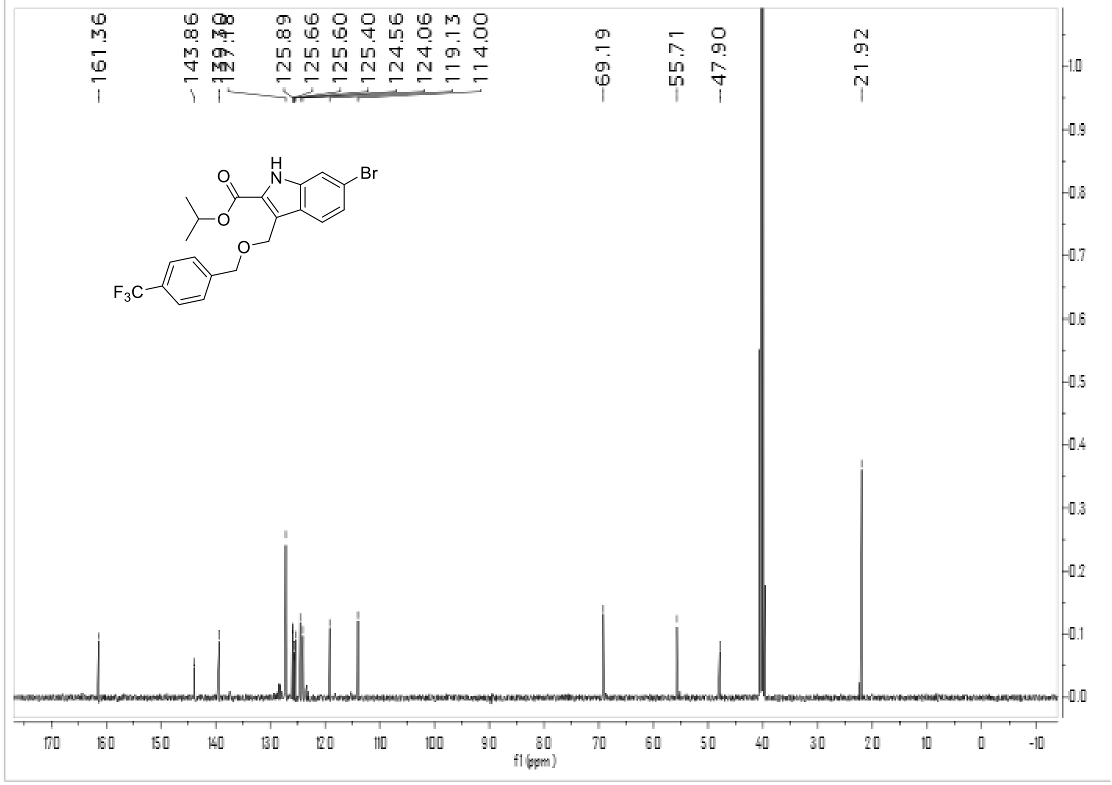

^{13}C NMR spectrum of **15**

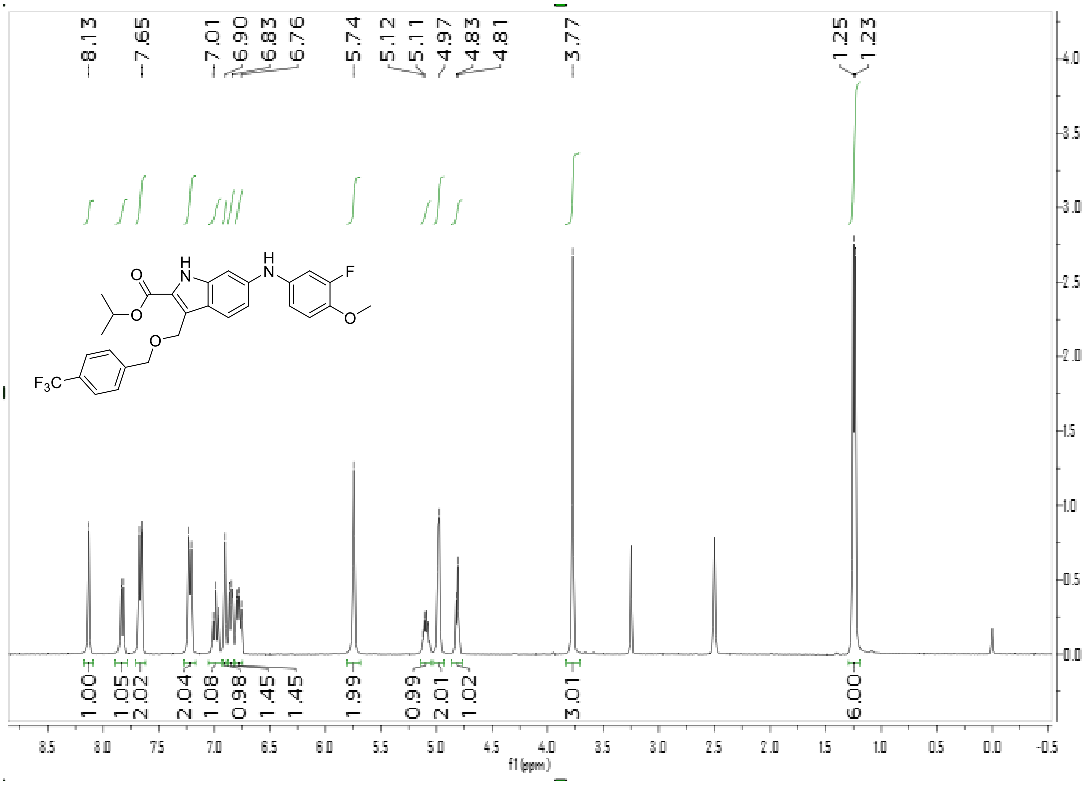

^{1}H NMR spectrum of **16a**

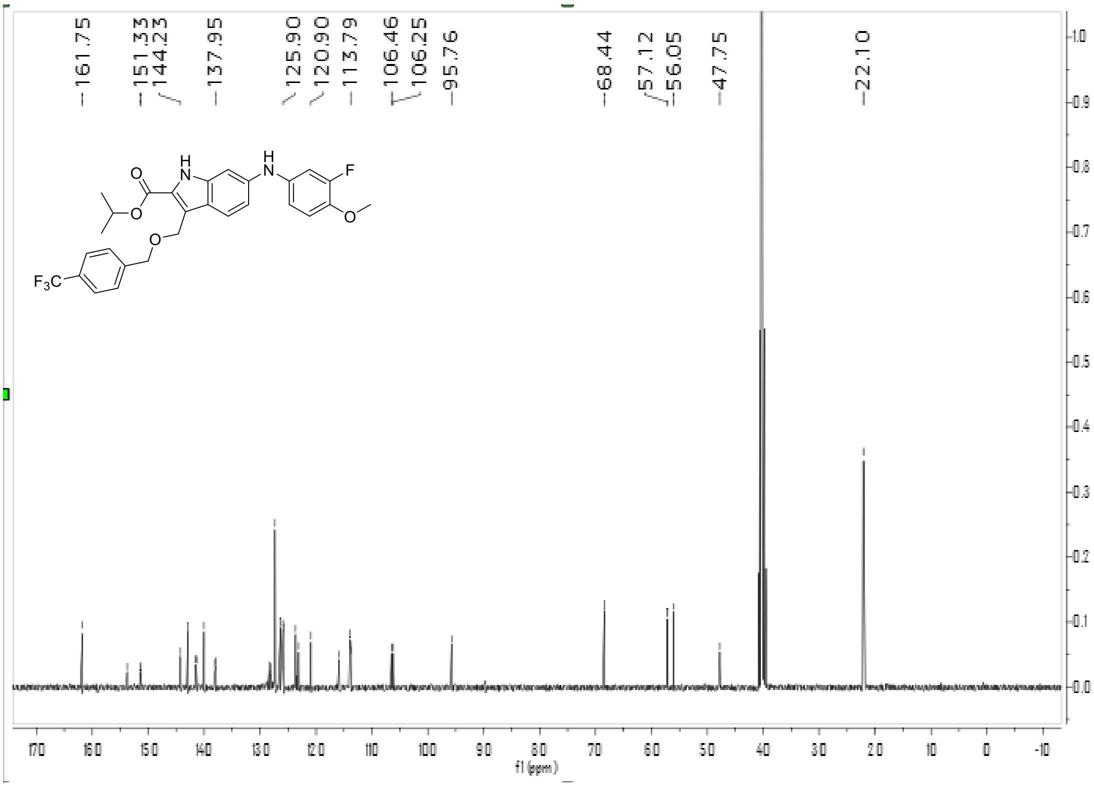

^{13}C NMR spectrum of **16a**

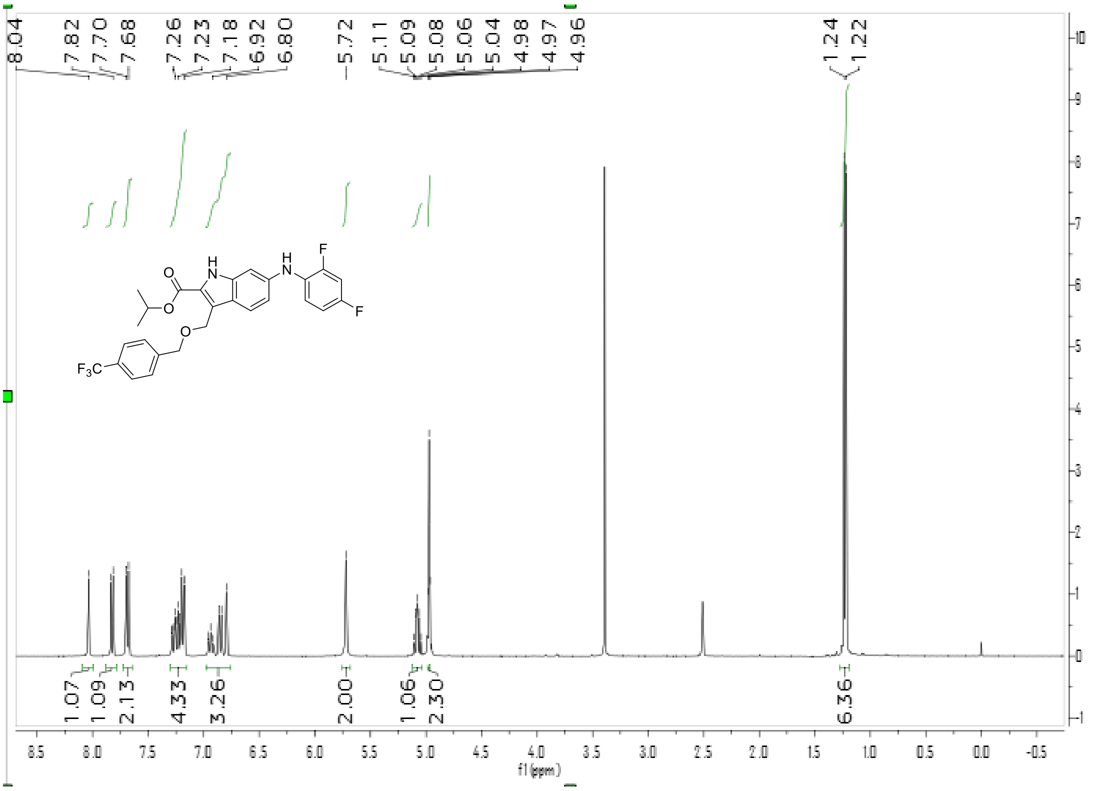

^{1}H NMR spectrum of **16b**

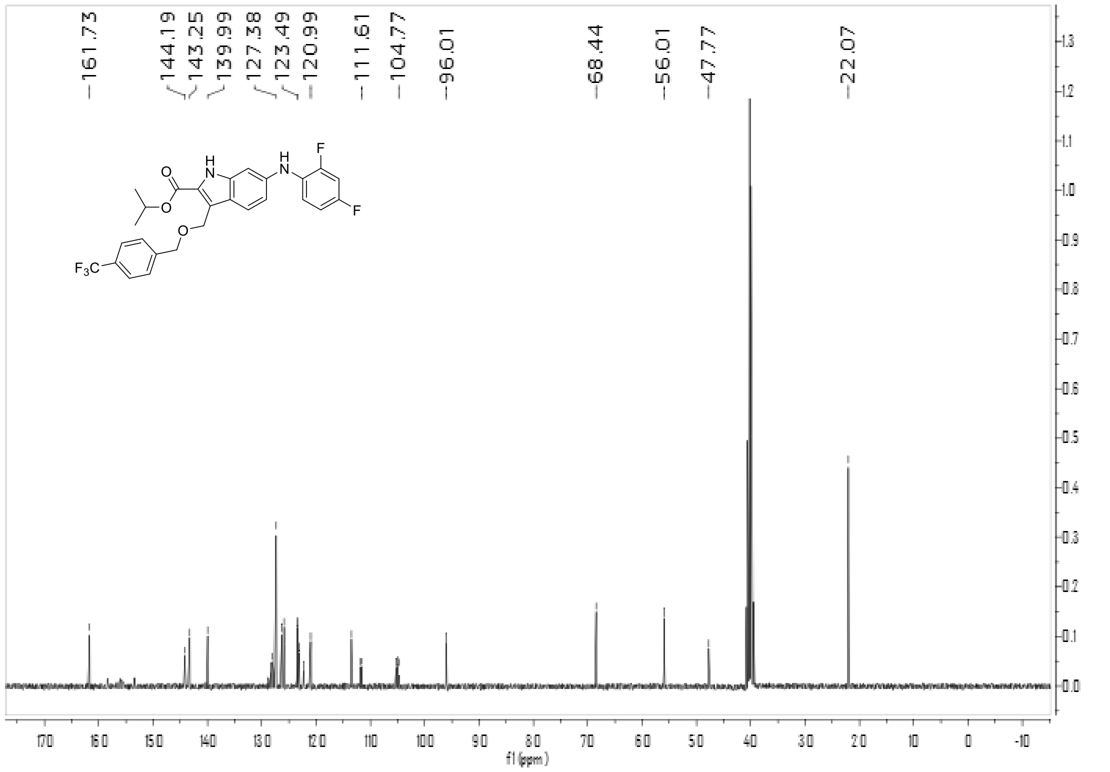

^{13}C NMR spectrum of **16b**

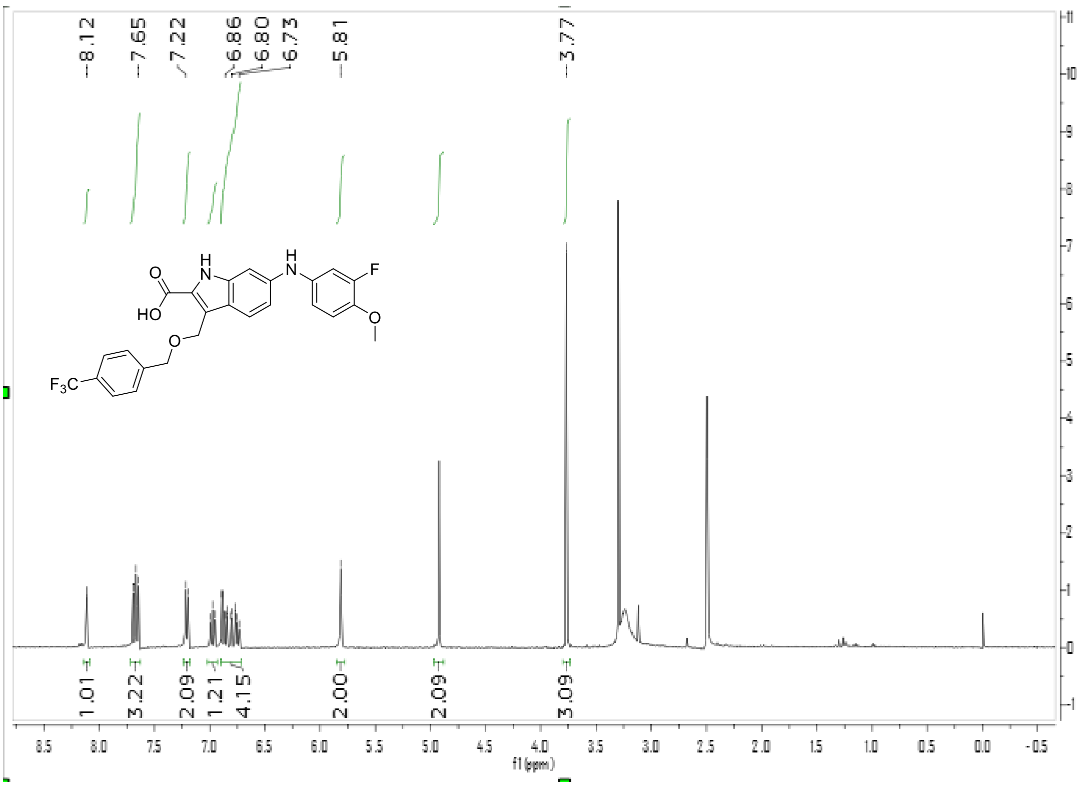

^{1}H NMR spectrum of **17a**

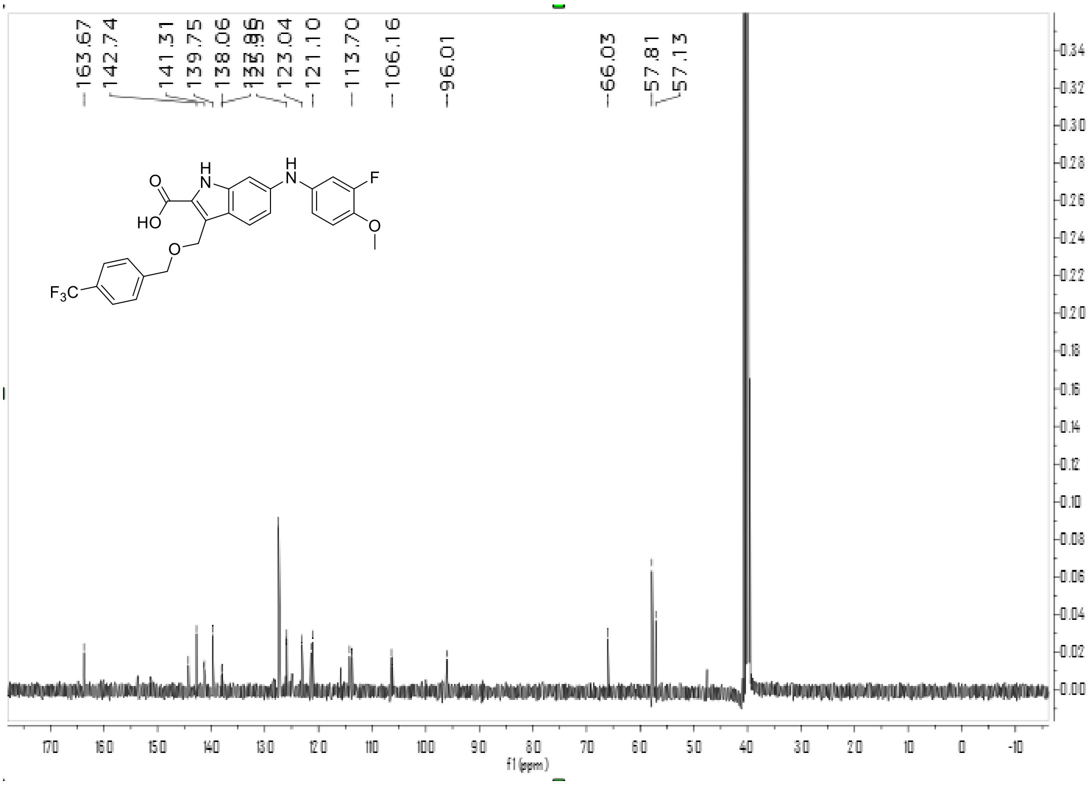

^{13}C NMR spectrum of **17a**

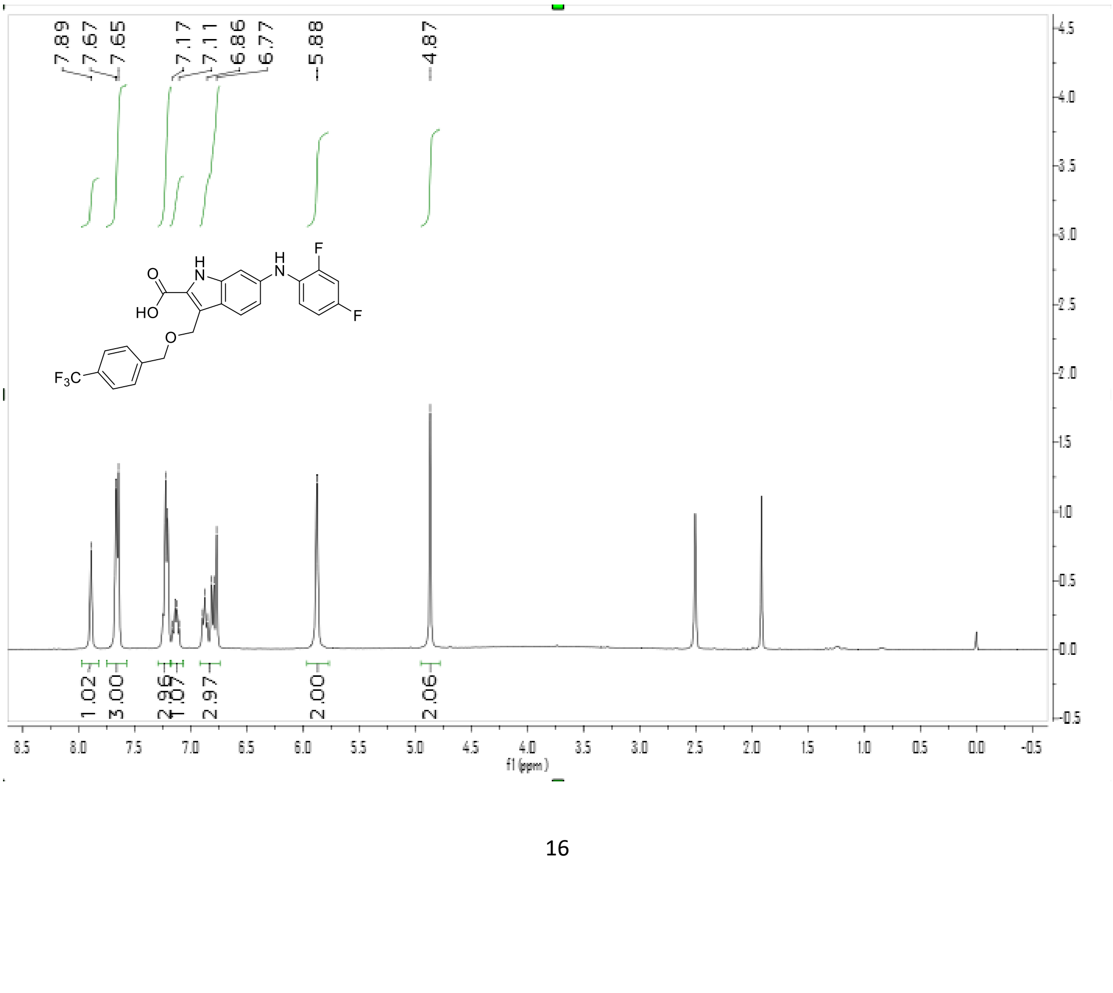

^{1}H NMR spectrum of **17b**

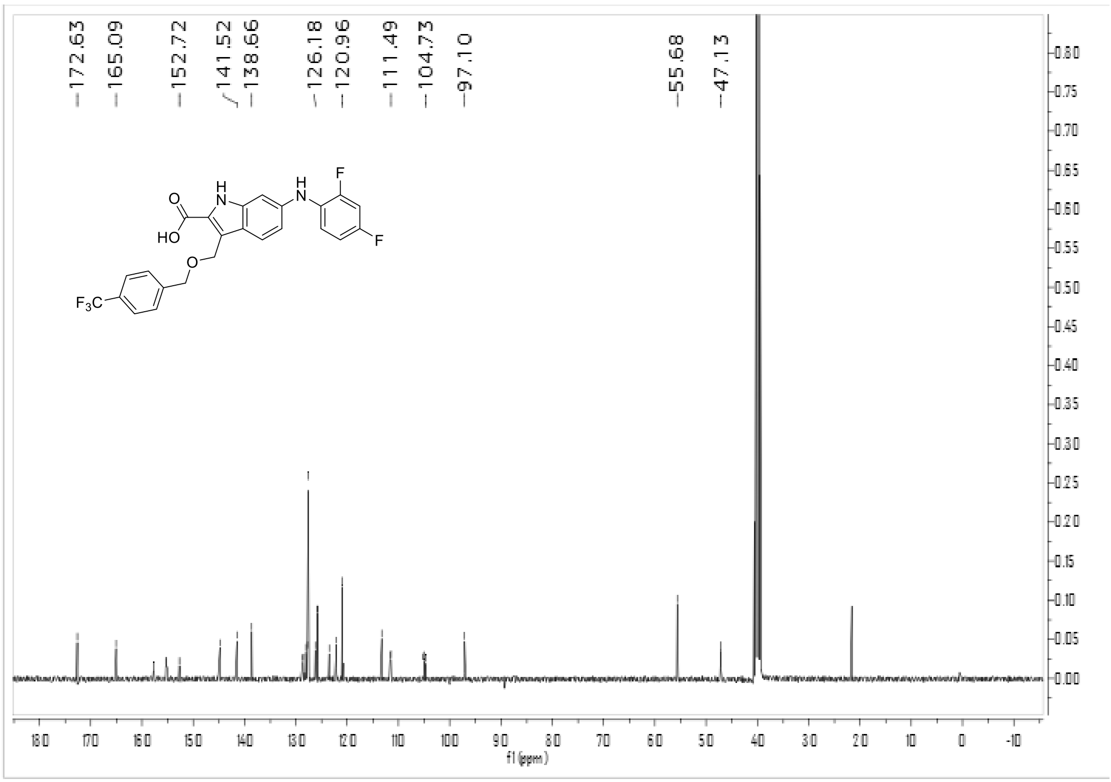

^{13}C NMR spectrum of **17b**

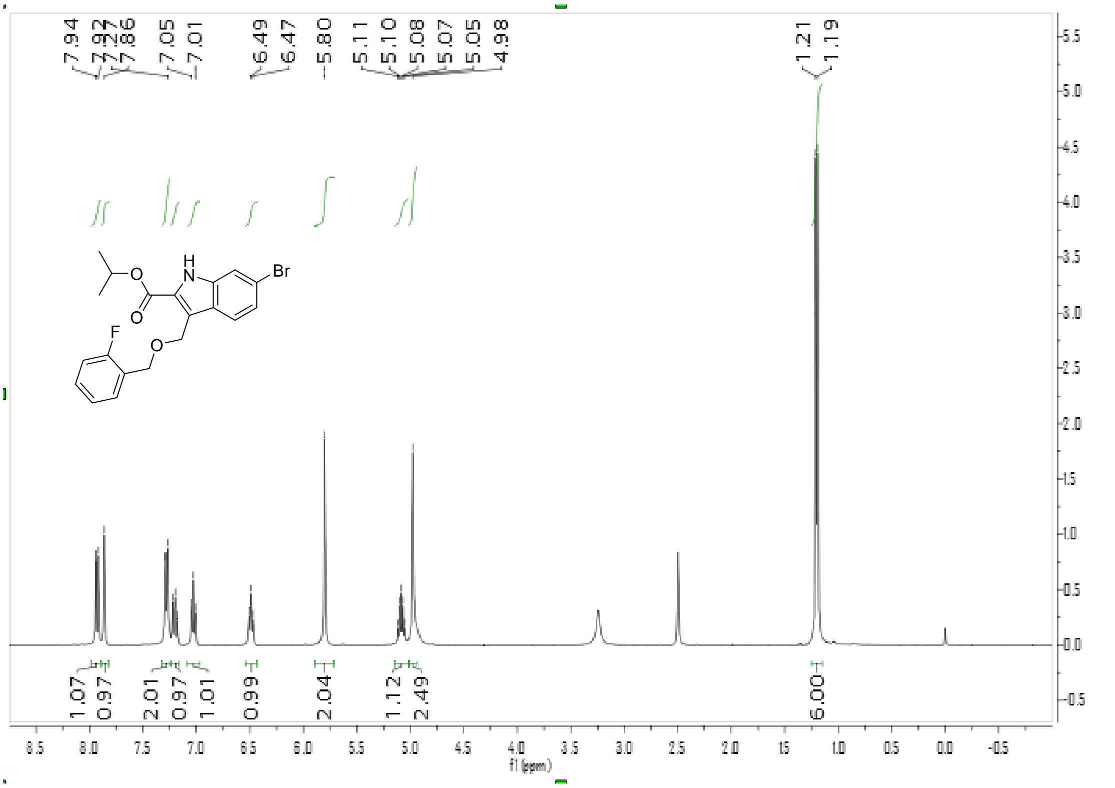

^{1}H NMR spectrum of **18**

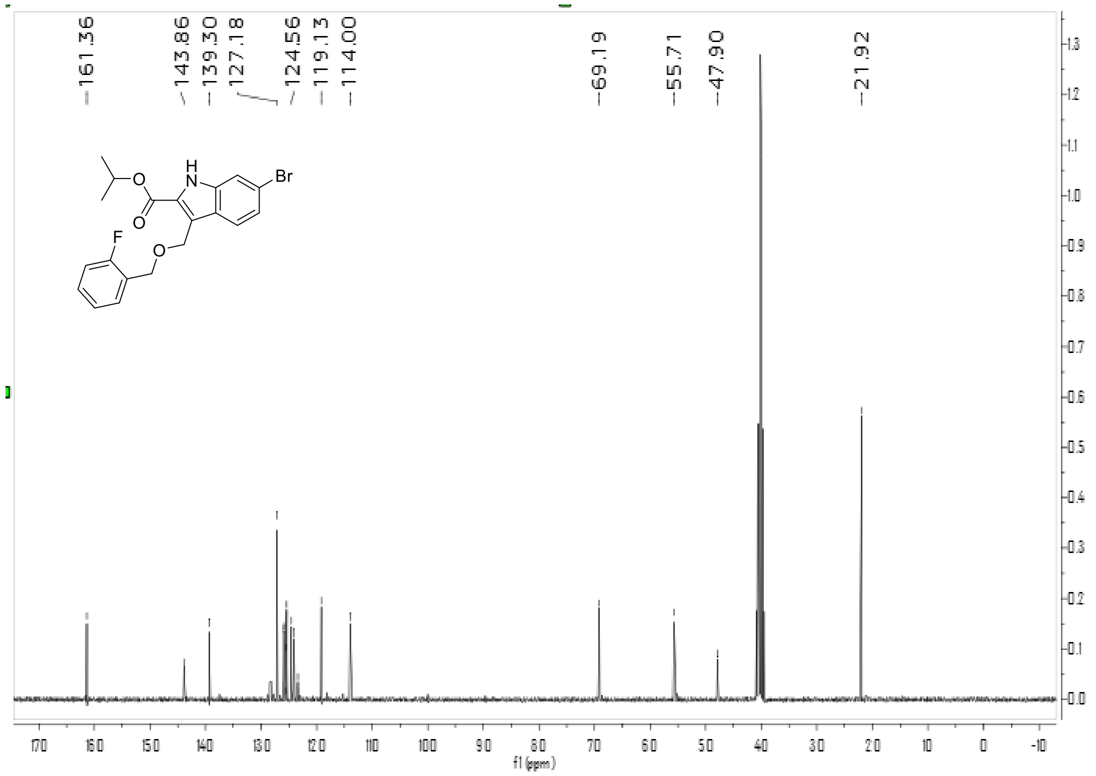

^{13}C NMR spectrum of **18**

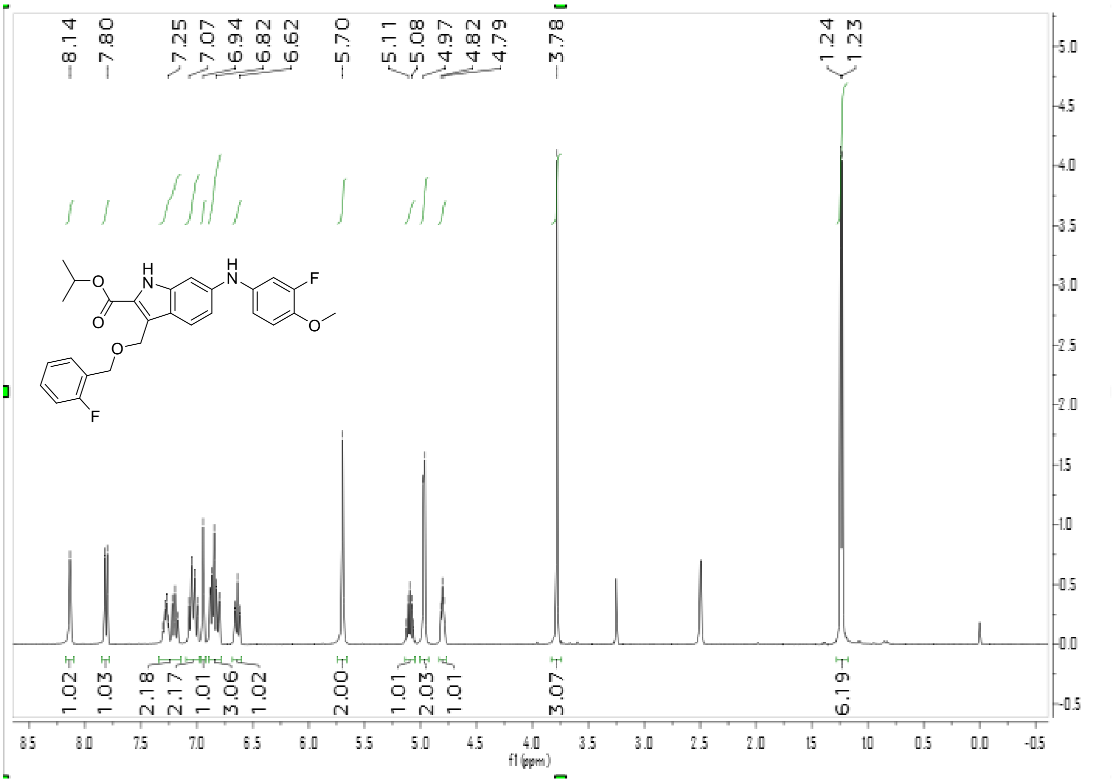

^{1}H NMR spectrum of **19a**

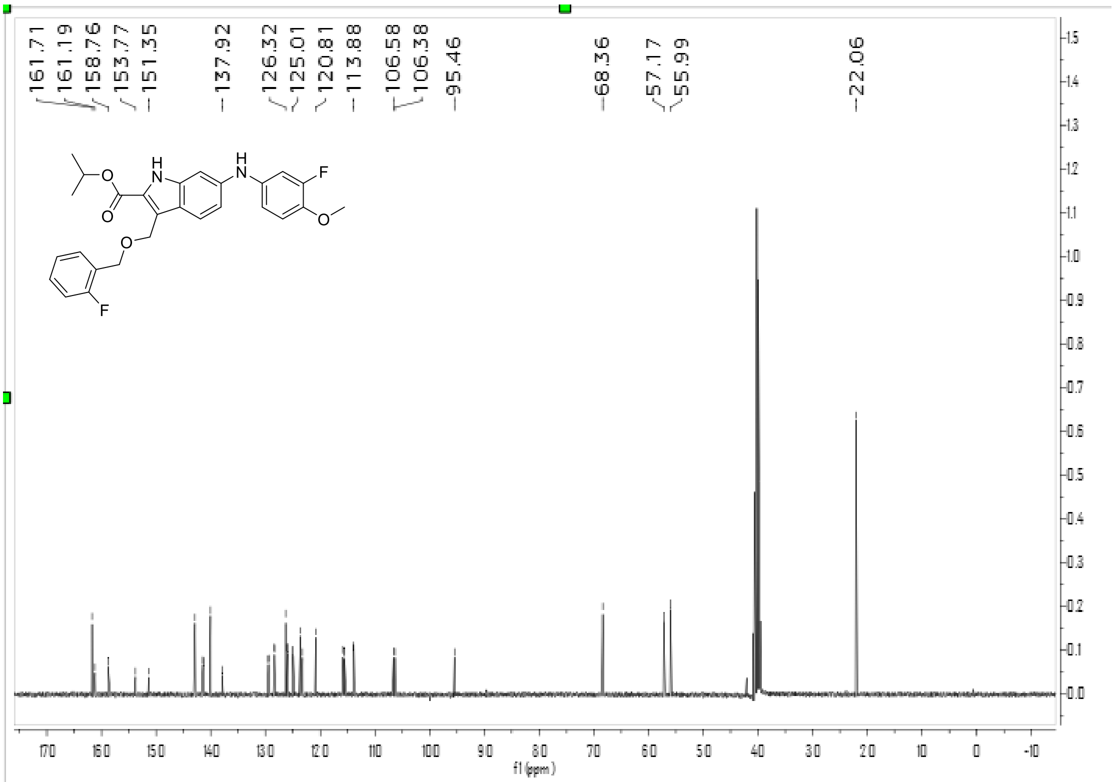

^{13}C NMR spectrum of **19a**

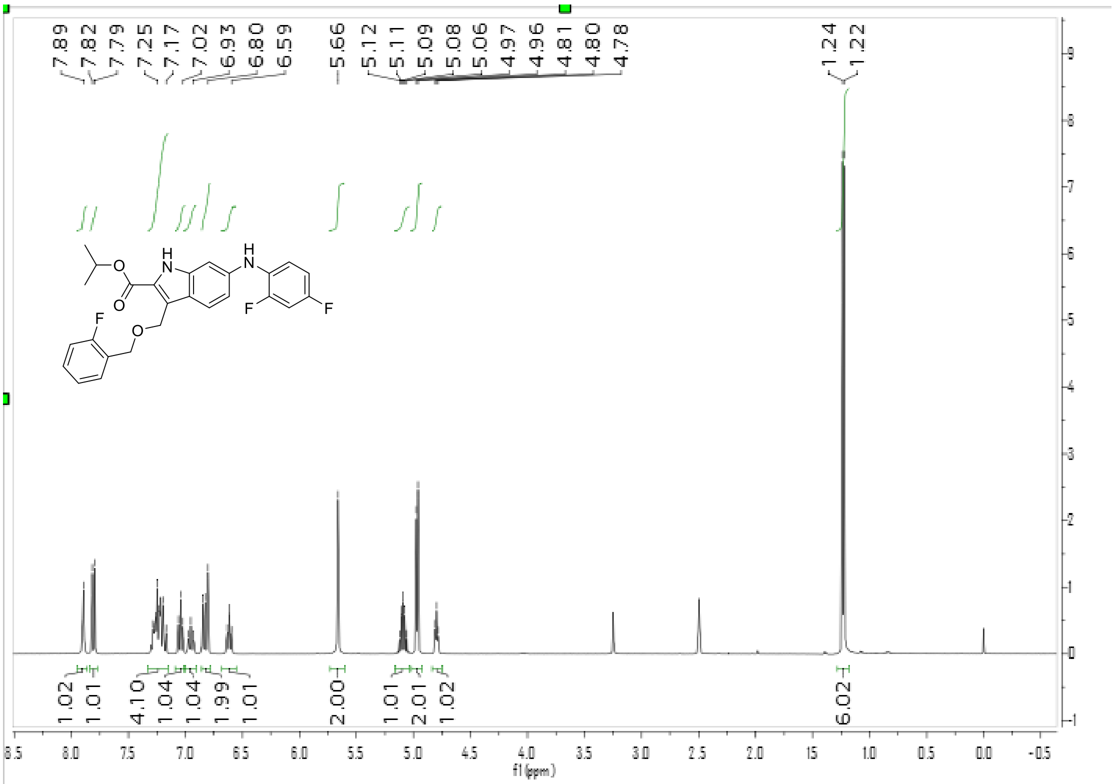

^{1}H NMR spectrum of **19b**

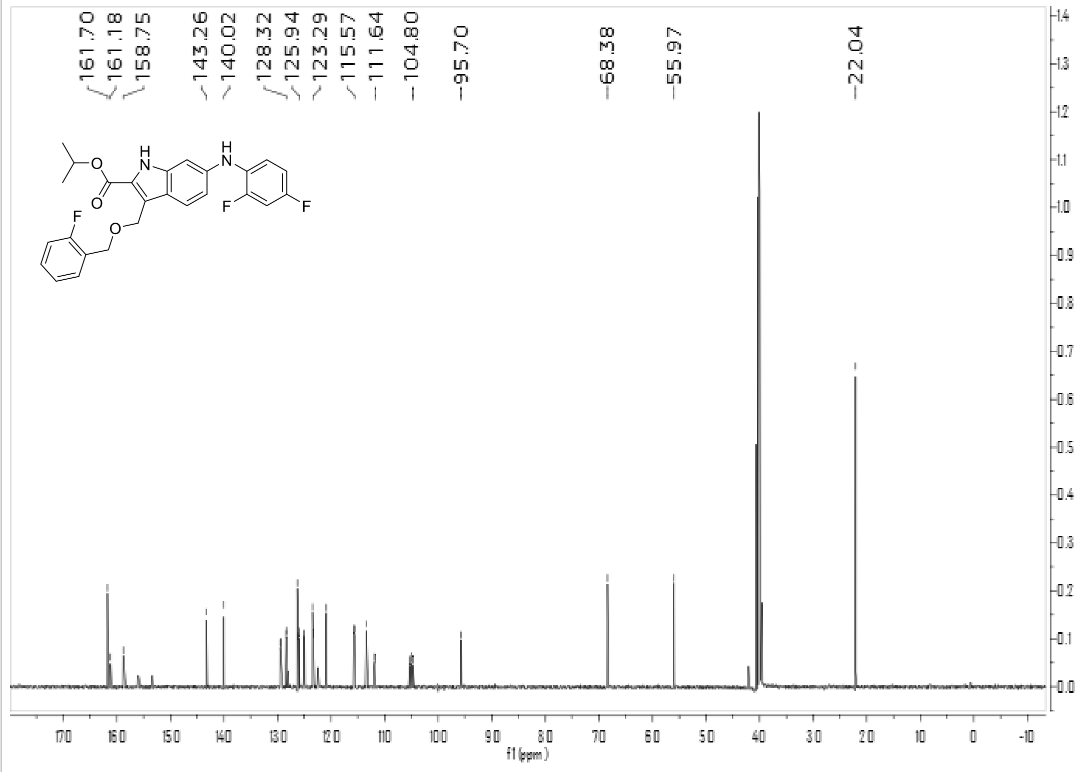

^{13}C NMR spectrum of **19b**

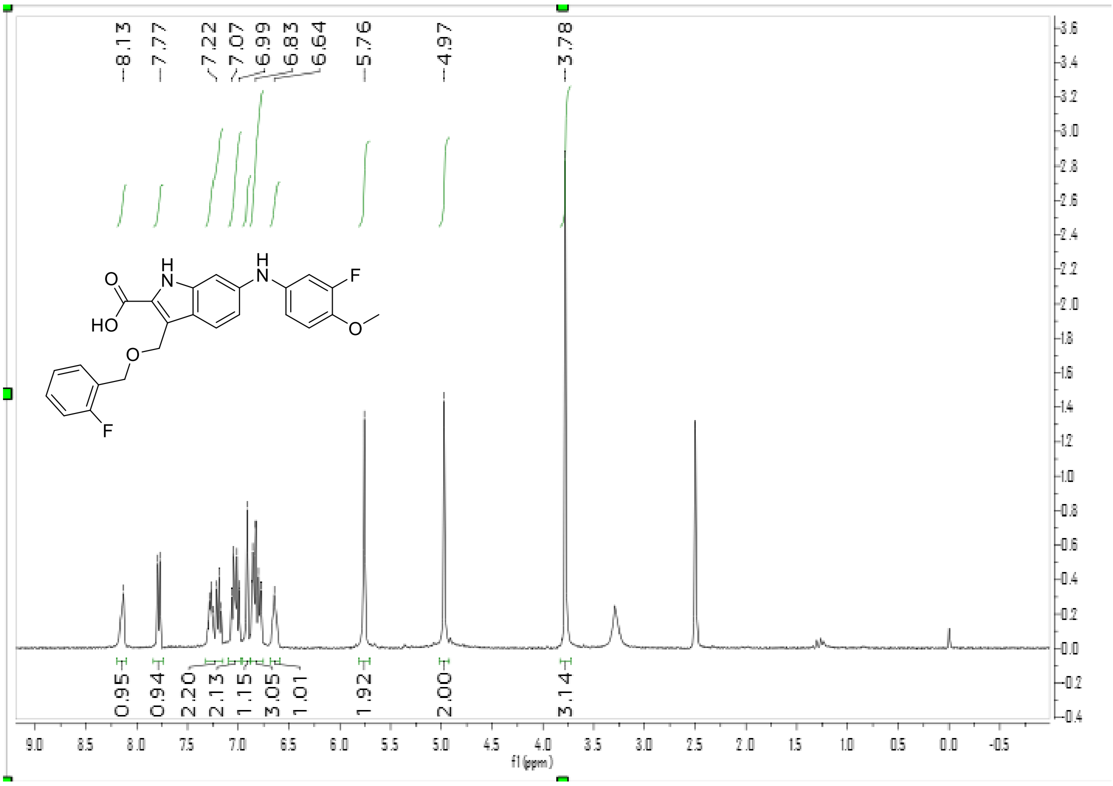

^{1}H NMR spectrum of **20a**

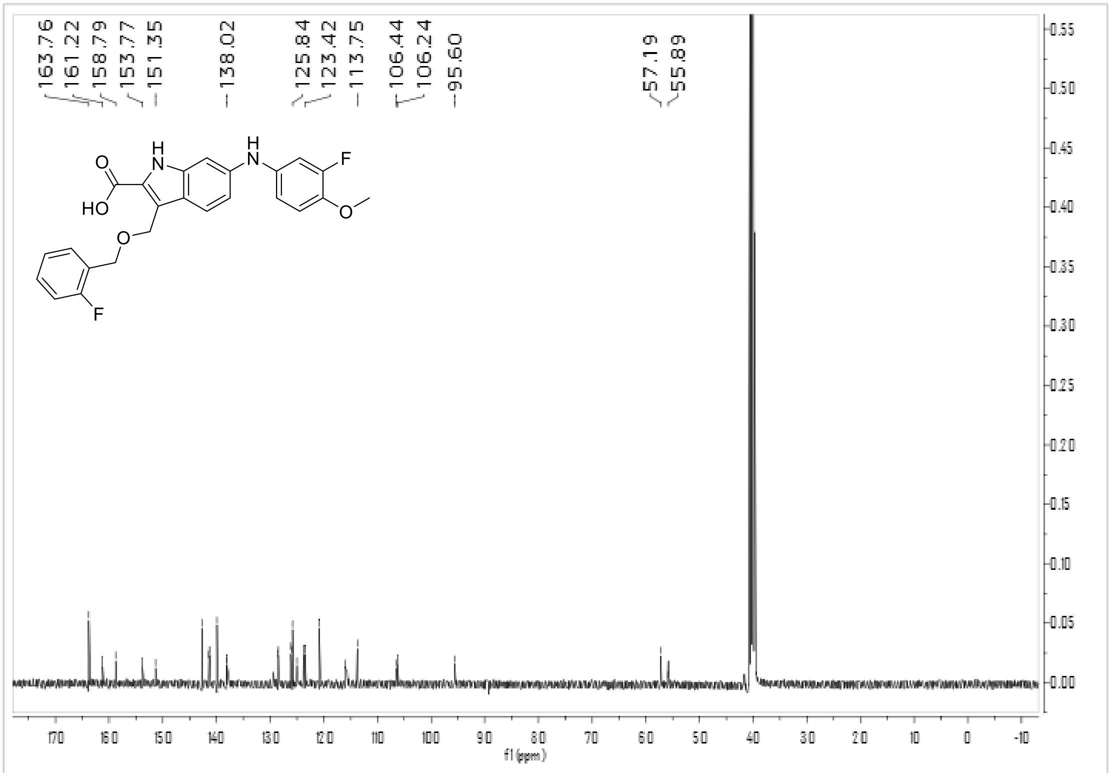

^{13}C NMR spectrum of **20a**

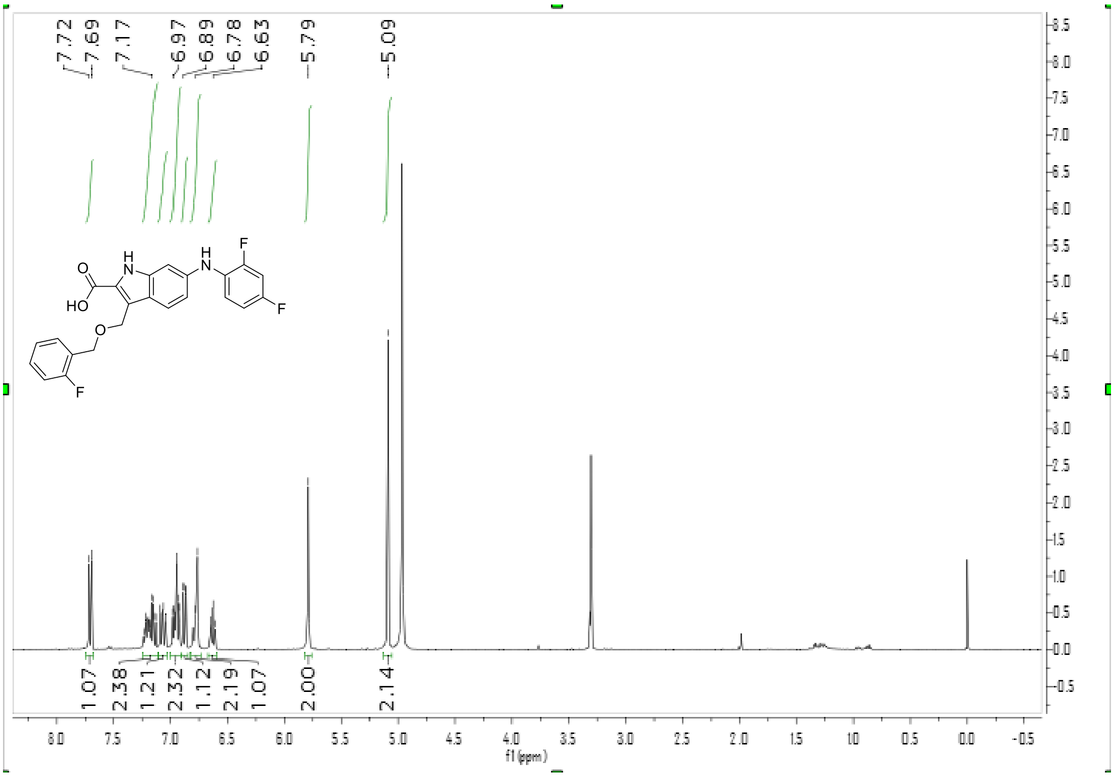

^{1}H NMR spectrum of **20b**

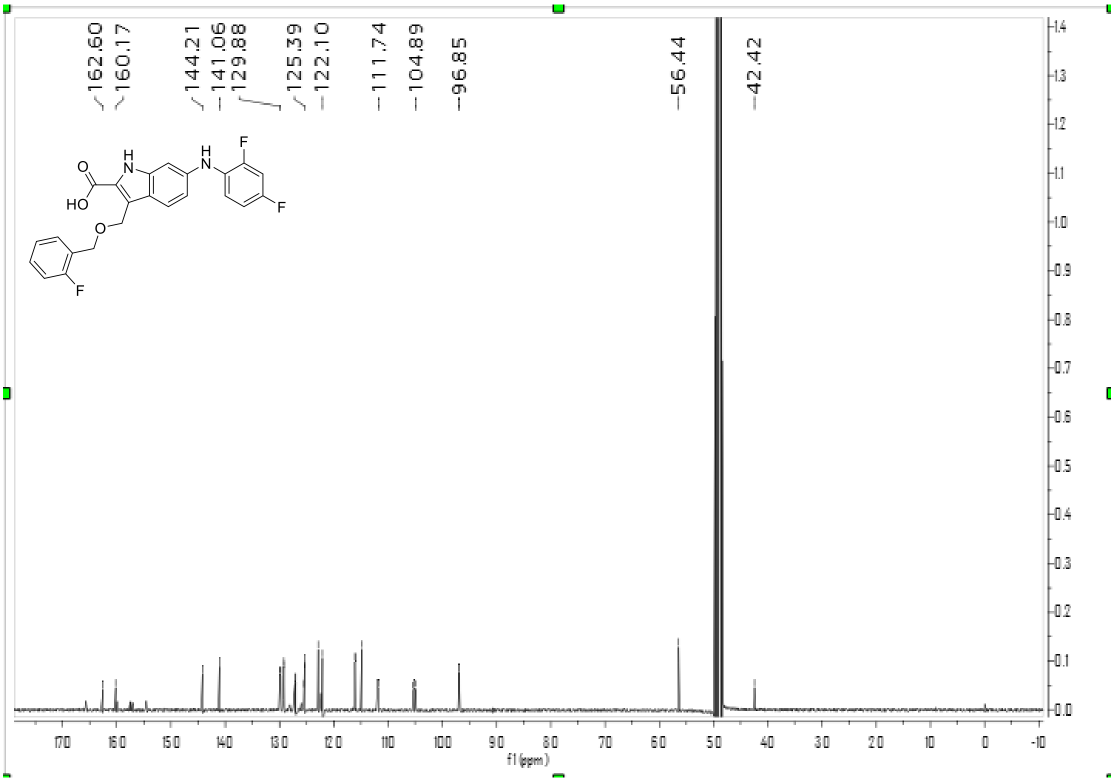

^{13}C NMR spectrum of **20b**

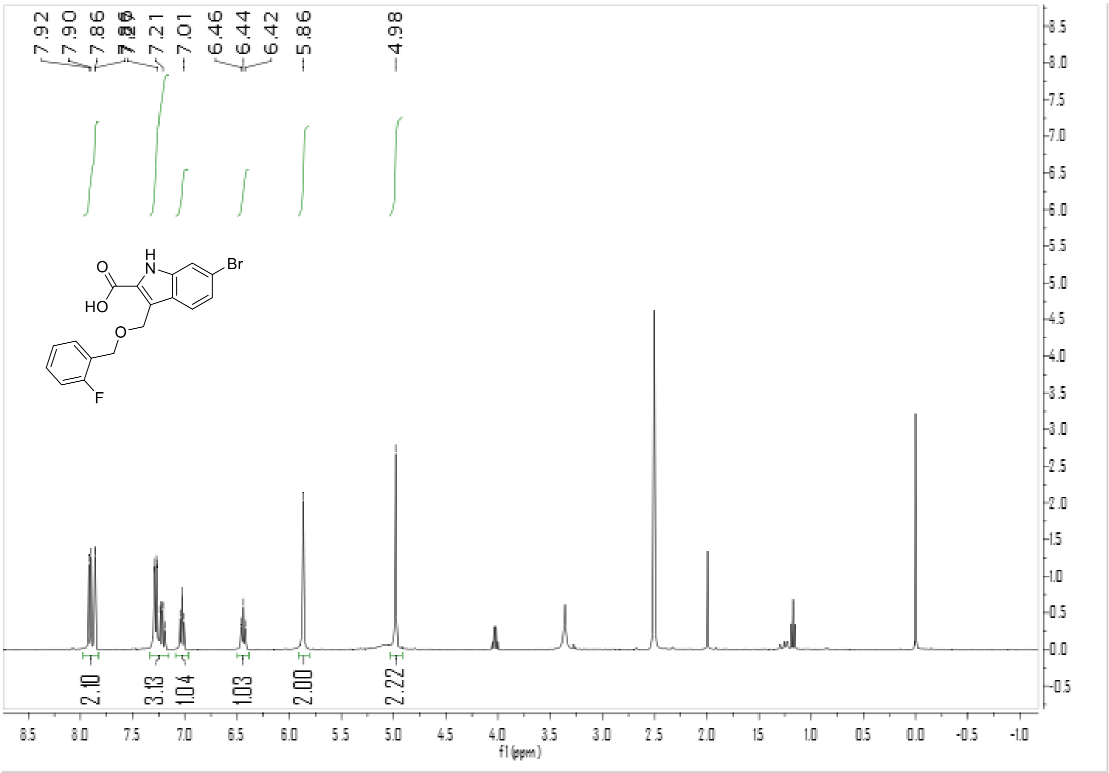

^{1}H NMR spectrum of **21**

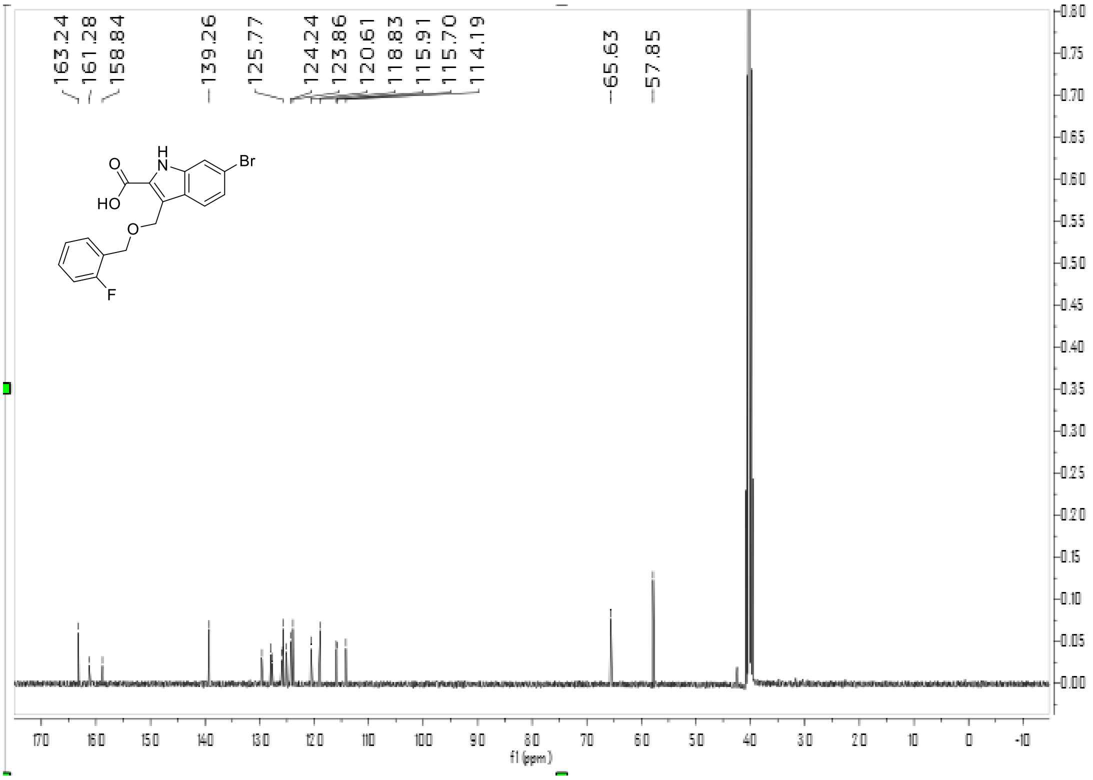

^{13}C NMR spectrum of **21**

3. IC_{50} and CC_{50} curves of target compounds

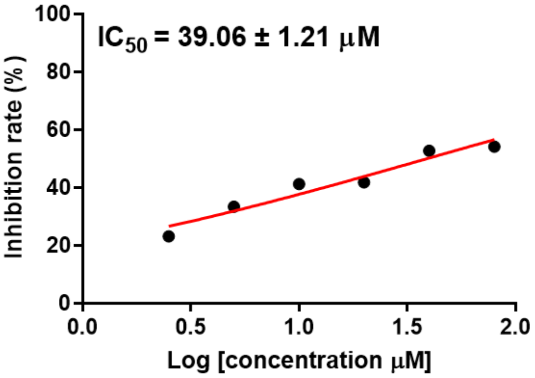

IC_{50} of compound **1**

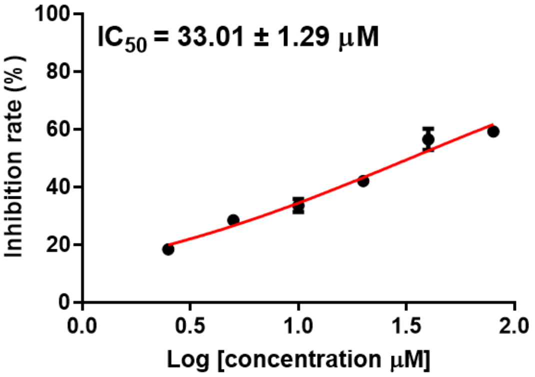

IC_{50} of compound **2**

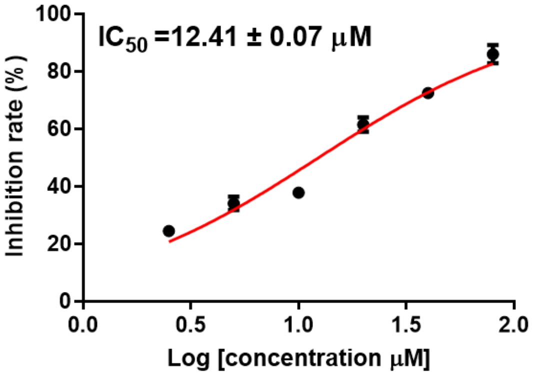

IC_{50} of compound **3**

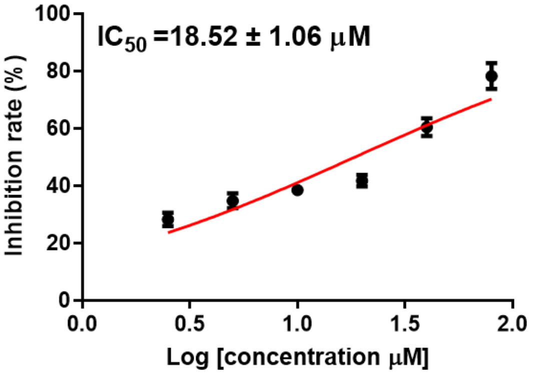

IC_{50} of compound **4**

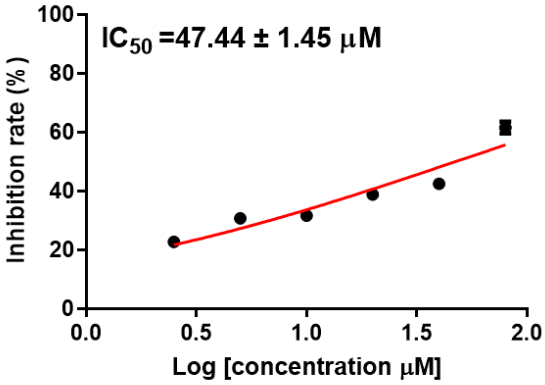

IC_{50} of compound **5**

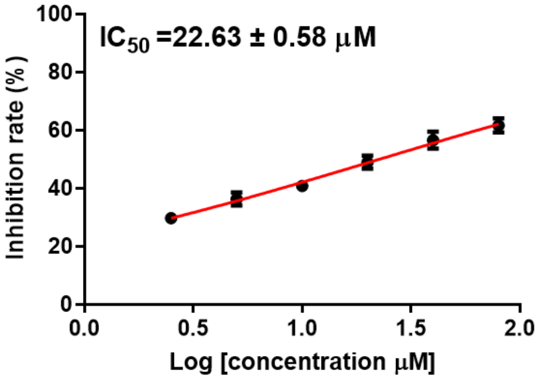

IC_{50} of compound **6**

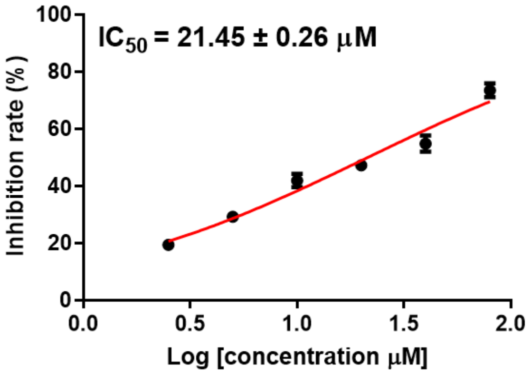

IC$_{50}$ of compound **7**

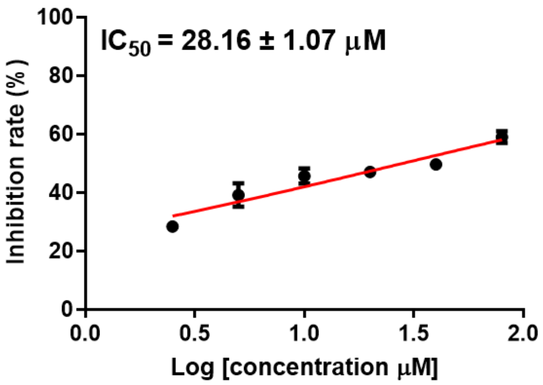

IC$_{50}$ of compound **8**

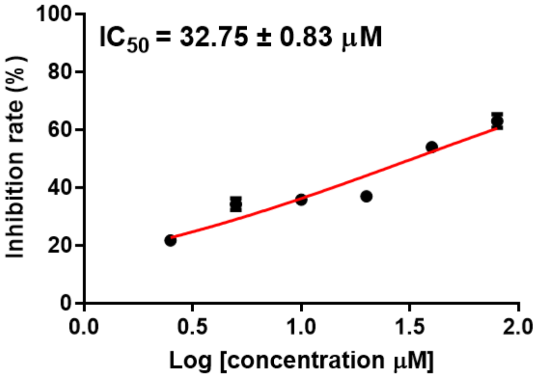

IC$_{50}$ of compound **9**

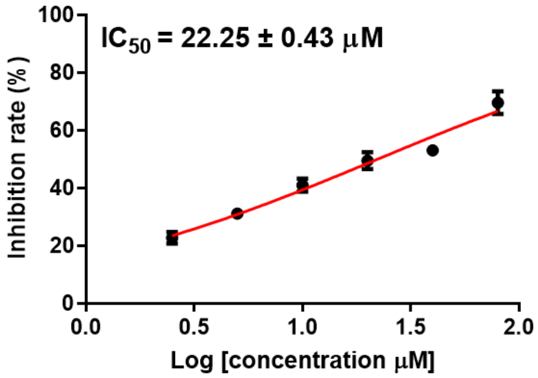

IC$_{50}$ of compound **10**

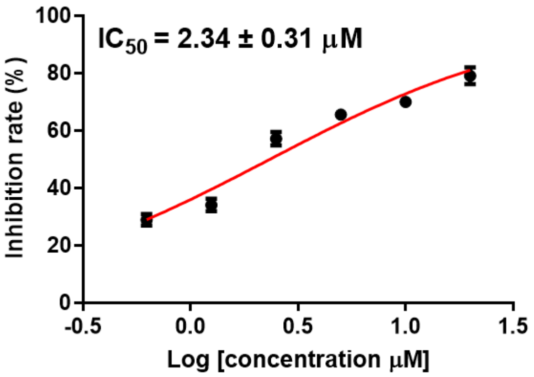

IC$_{50}$ of compound **15**

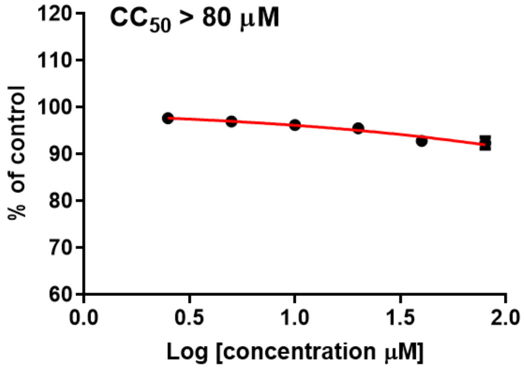

CC$_{50}$ of compound **15**

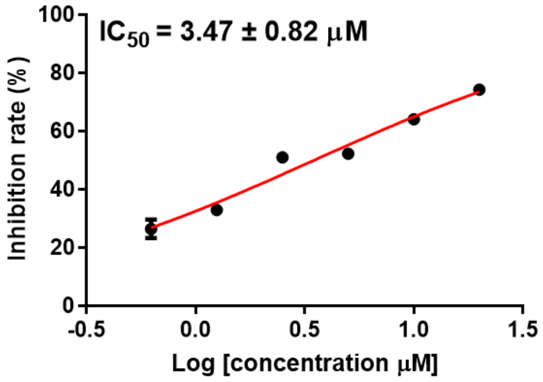

IC50 of compound **16a**

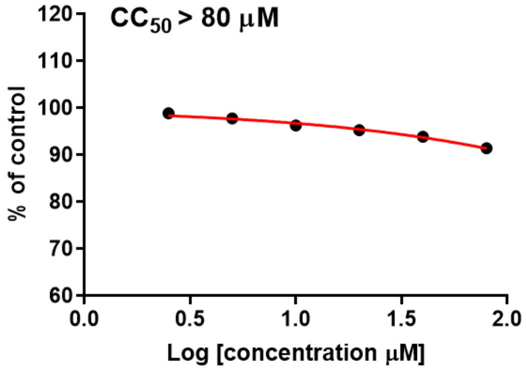

CC50 of compound **16a**

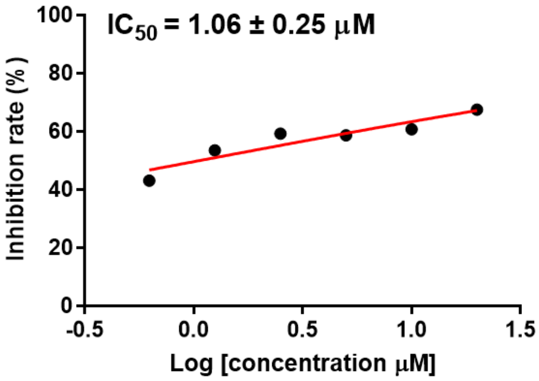

IC50 of compound **16b**

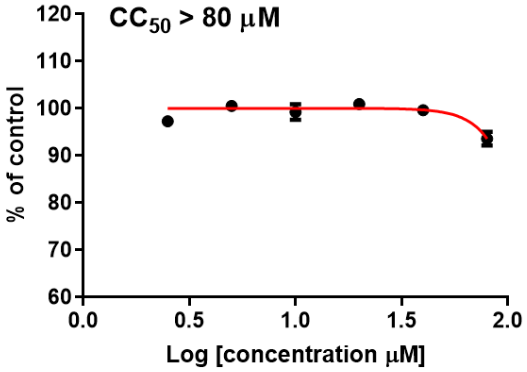

CC50 of compound **16b**

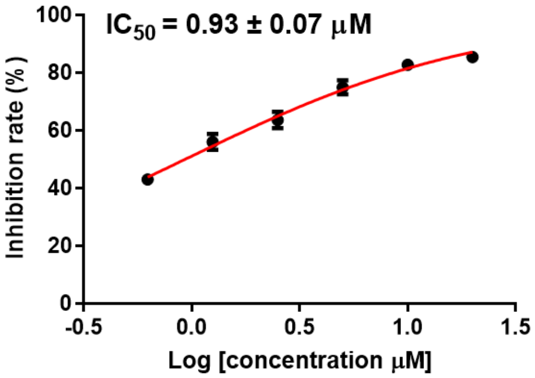

IC50 of compound **17a**

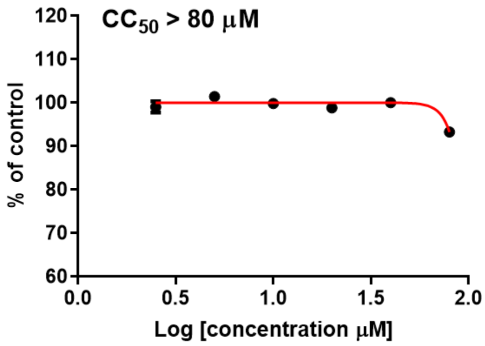

CC50 of compound **17a**

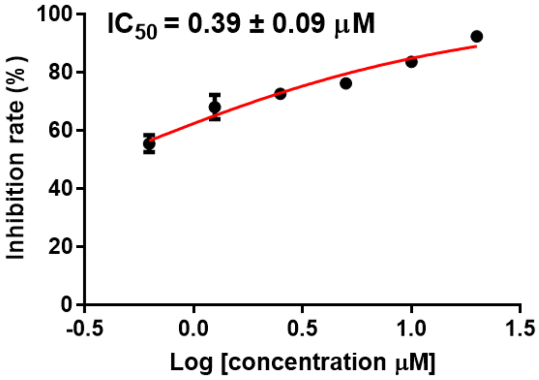

IC50 of compound **17b**

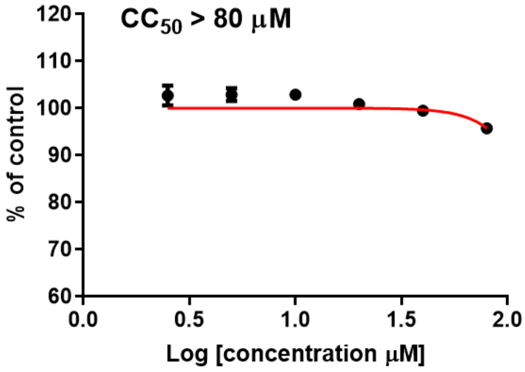

CC50 of compound **17b**

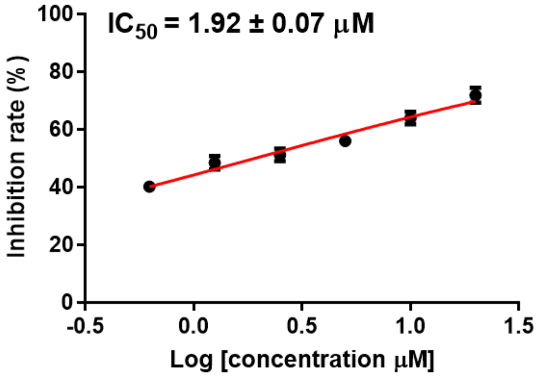

IC50 of compound **18**

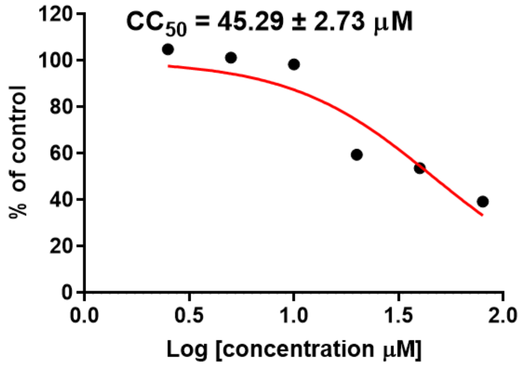

CC50 of compound **18**

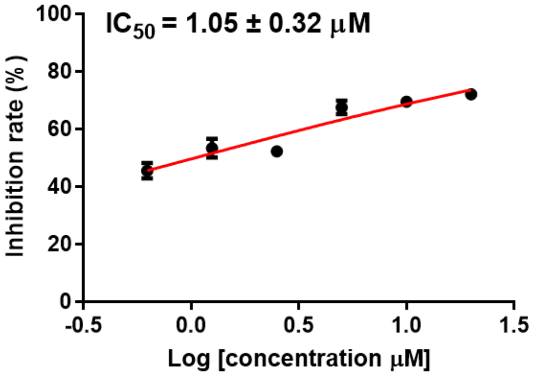

IC50 of compound **19a**

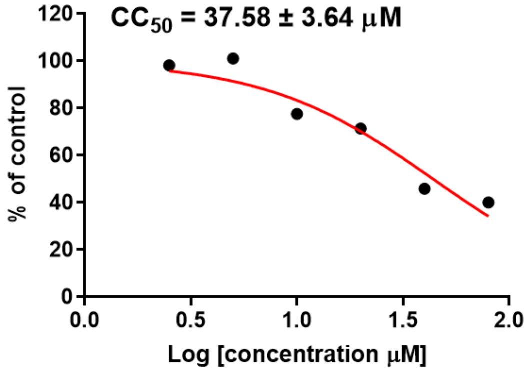

CC50 of compound **19a**

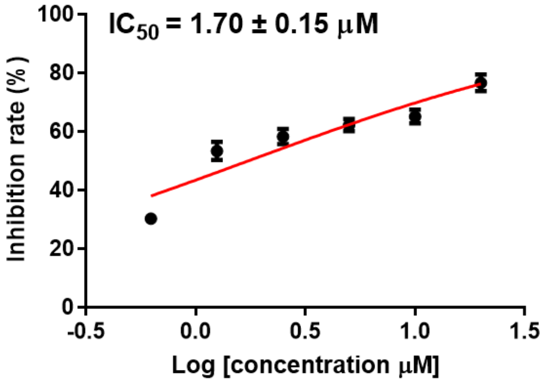

IC50 of compound **19b**

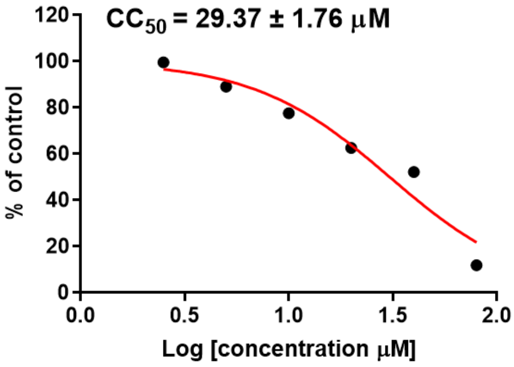

CC50 of compound **19b**

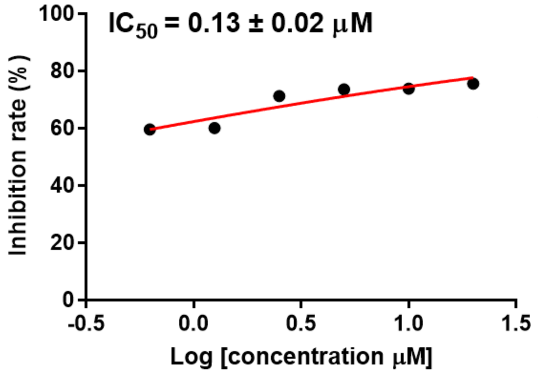

IC50 of compound **20a**

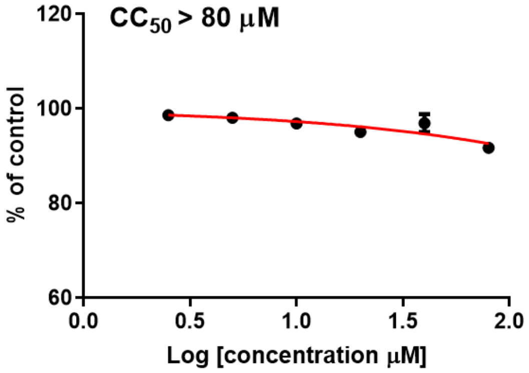

CC50 of compound **20a**

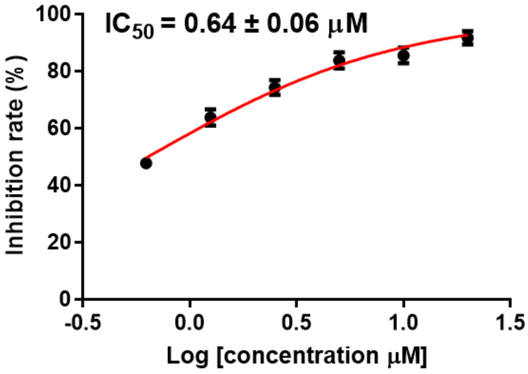

IC50 of compound **20b**

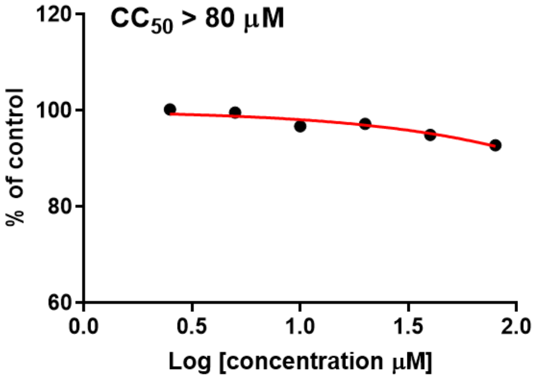

CC50 of compound **20b**

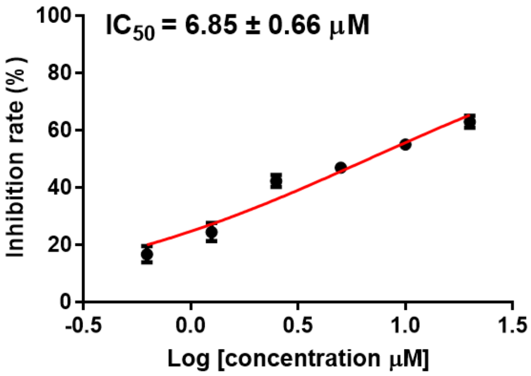

IC50 of compound **21**

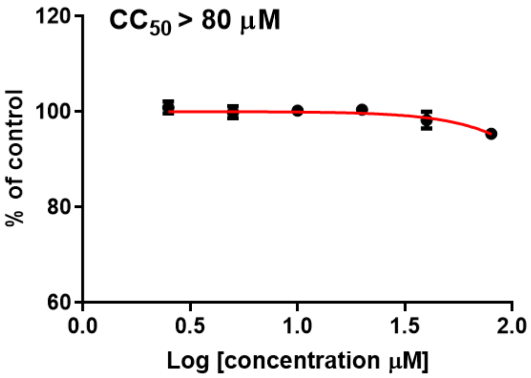

CC50 of compound **21**
